# Transition metals doped effects for the crystal stabilization of the cerium oxides with the first principle calculation

**DOI:** 10.1038/s41598-022-14180-3

**Published:** 2022-06-16

**Authors:** Takaki Nishimura, Tatsuya Kodama, Sakane Genta, Tomohiko Ishii

**Affiliations:** 1 Department of Advanced Materials Science, Graduate School of Engineering, Kagawa University, 2217-20 Hayashi-cho, Takamatsu, Kagawa 761-0396 Japan; 2 Department of Engineering, Niigata University, 8050 Igarashi 2no-cyo, Nishi-ku, Niigata, 950-2181 Japan; 3 Department of Chemistry, Faculty of Science, Okayama University of Science, 1-1 Ridaicho, Kita-ku, Okayama, 700-0005 Japan

**Keywords:** Hydrology, Energy science and technology, Materials science

## Abstract

In recent years, hydrogen energy has been attracting attention, and the hydrogen gas production using solar thermal energy has been conducted. The studies of Kodama et al. were reported that the cyclic reaction can efficiently produce the hydrogen gas through a two-step thermal redox reaction with the cerium oxide. The transition metal doping into the cerium oxide improved the reaction efficiency. We considered the doping effect on the thermal two-step redox reaction. As a result of the calculation by the DV-Xα method, it was clarified that the doped cerium oxide becomes a strong bond, the large BOP value without changing the ceria crystal structure in the two-step thermal redox reaction. The theoretical calculation results corresponded to the reaction efficiency improvement of the thermal reaction in experimental results.

In recent days, the global hydrogen gas production is about 700 billion Nm^3^, enough to supply the fuels for more than 600 million fuel cell vehicles. However, about half of the hydrogen gas is made from natural gas. Nearly 1/3 of the hydrogen is made of crude oil in the refineries. And most of the hydrogen gas is consumed in the refineries^1^. From this background, many studies have also been reported on the sustainable production of the hydrogen energy from solar thermal energy, focusing on solar energy that can be used permanently^2–5^. Solar energy irradiates energy from the sun to the earth, typed by renewable energy. It is equivalent to about 4 million EJ (1 EJ = 1018 J) per year^6^.

The amount of energy theoretically extracted could be about 19,000 EJ, and the amount of energy that can be technically extracted is estimated to be only about 1900 EJ. It is also estimated to consume just about 20 EJ energy per year in human living. If we could convert a vast amount of solar energy into other energy, we could get enough energy for our living^7^. So, it makes sense to use solar energy for hydrogen energy production. In addition, various studies have been conducted on the supply chain, such as the transport and operation^8^. Hydrogen gas is also transported by tanker and truck after being converted into liquid hydrogen or liquid fuels such as methanol, ammonia, and methylcyclohexane (MCH). The basis for producing hydrogen energy is also making significant progress, so the importance of hydrogen energy production is increasing^1,9,10^. In laboratory experiments^11–16^, many hydrogen gas production demonstration experiments using beam down solar concentrators have been conducted^17–20^, and it is expected that commercialization of hydrogen gas production will become possible.

In hydrogen gas production, a two-step thermal redox reaction is used. The two-step thermal redox reaction is a cyclic redox reaction consisting of a reduction reaction (1st step: Eq. 1) with oxygen desorption at high temperatures (> 1000 ℃) and an oxidation reaction (2nd step: Eq. 2) with oxygen adsorption at low temperatures (< 1000 ℃). In the thermal oxidation reaction (2nd step), it is possible to decompose water molecules and efficiently produce hydrogen gas under high temperature steam^14^.

1 $${\text{1st}}\,\,\,{\text{step}}: {\text{M}}_{{\text{x}}} {\text{O}}_{{\text{y}}} \to {\text{ M}}_{{\text{x}}} {\text{O}}_{{{\text{y}} - \delta }} + \, \delta /{\text{2O}}_{{2}}$$

2 $${\text{2nd}}\,\,{\text{step}}: {\text{M}}_{{\text{x}}} {\text{O}}_{{{\text{y}} - \delta }} + \, \delta {\text{H}}_{{2}} {\text{O }} \to {\text{ M}}_{{\text{x}}} {\text{O}}_{{\text{y}}} + \, \delta {\text{H}}_{{2}}$$

In addition to the hydrogen production reaction, the thermal redox reaction can also be used for CO_2_ reforming methane^21,22^, so the application of the two-step thermal redox reaction is wide.

In the two-step thermal redox reaction, various metal oxides have been used as catalytic reaction ceramics to improve hydrogen gas production efficiency. Ehrhart et al*.* used hercynite (FeAl_2_O_4_) at the beginning of the hydrogen gas production study^23^. Wong et al*.* explored thermochemical heat storage (TCS) materials using thermal redox reactions in Co_3_O_4_/CoO and Mn_2_O_3_/Mn_3_O_4_^24^. Other research groups have also reported the two-step thermal redox reactions such as Mn_2_O_3_/Mn_3_O_4_, Co_3_O_4_/CoO, and CuO/Cu_2_O^25–29^. Kodama et al*.* have reported various hydrogen-producing materials using Fe_3_O_4_/c-YSZ, NiFe_2_O_4_^30^, NiFe_2_O_4_/m-ZrO_2_, Fe_3_O_4_/m-ZrO_2_^31^, Fe_3_O_4_/m-ZrO_2_/MPSZ^32,33^. Among these experiments, the research on cerium oxide has been promoted in recent years^34–37^. Cerium oxide is a type of lanthanoid oxide (CeO_2_) used as an Oxygen Storage Capacity (OSC) material. It was found to be applied to a catalyst and a thermal two‐step redox reaction.

In the hydrogen gas production studies, it was found that cerium oxide increases the hydrogen gas production efficiency and the redox reaction cyclability^38^. Furthermore, doping transition metals into cerium oxide showed further the efficiency, and cyclability has also been reported^39,40^. Cho et al*.* have investigated the doped ceria as an operational test^41^. A 3 kW sun-simulator irradiated the redox reactive foam devices. Among other things, Jacot et al*.* have reported good experiments and theoretical research on the CO_2_ reduction and water splitting^42^. The report showed the various doped cerium oxide. Hf-, Zr-, and Ta-doped cerium oxides were reported to have a high reaction efficiency. Many studies have been reported using electrolysis as well as thermodynamic hydrogen generation^43,44^. Nowadays, Zhao et al*.* showed the suitable materials for the electrolysis, the HER catalysts^45^.

Although it is clear from the experimental facts that cerium oxide-based materials are effective, the following point should be more reported like the paper (Jacot et al*.*)^42^.

(i) The hydrogen gas production properties of doped cerium oxide exceed those of pure cerium oxide.

As a specific point, we decided to focus on the stability of the crystal structure. It is the bond strength between the metal ions and the oxygen ions in the cerium oxide. The thermal reduction reaction means that the oxygen ions in cerium oxide are defective, while the thermal oxidation reaction is that the oxygen is taken into the cerium oxide crystal. So, the oxygen atoms adsorption and desorption are the critical matter during the thermal redox reaction. In addition, the experimental fact is that the transition metal doping improves the reaction efficiency and cyclability. It was expected that the transition metal doping into cerium oxide changes to chemical bonds suitable for the redox reaction cycle. We tried to solve question (i) based on these hypotheses.

This study aims to theoretically explain the experimental facts concerning the above question (i) contents by using DV-Xα molecular orbital calculation. The DV-Xα method was developed by D. E. Ellis (Northwestern University) and H. Adachi (Kyoto University)^47–51^. The self-consistent field method (SCF method) was proposed by Hartree in 1928 and included the Hartree–Fock-Slater method proposed by J. C. Slater^52^. The electronic potential proposed by Slater is called “Xα potential,” and the DV-Xα method is another name for the Hartree–Fock-Slater method. The DV-Xα method has the advantage of numerically evaluating the electronic state. Therefore, accurate calculation results can be obtained for the *d-* or *f-*orbitals of the metal atoms. Due to the above advantages, the DV-Xα method is used in the theoretical calculations for cluster models of the doped cerium oxide.

## Result and discussion

### Evaluation of BOP

In the metal-doped ceria, it was suggested that the stability of the ceria crystal structure was improved by doping the transition metals, as described in the introduction. We used the BOP value as a parameter of bond strength to discuss the stability of the ceria crystal structure. If the BOP values become larger, there is more overlap in the wave functions between the two atoms, and the bond becomes a strong bond. The detail of the calculation is described at the last of the paper. In this study, to discuss the stabilization of the metal-doped crystal structure, we consider the bond strength between the doped metal atom (M) and the surrounding oxygen atoms in the M@Ce_12_O_8_^36+^ cluster model. We discuss the stability of the crystal structure in the doped ceria by comparing the BOP values between the M–O two atoms.

At first, we focused on the doping of Mn, Fe, Co, and Ni as the *3d*-orbital transition metals to discuss the doped ceria with Mn, Fe, Co, and Ni reported by Kodama et al*.*. We also report the theoretical calculation results of the ceria with Ti, V, Cr, Mn, Fe, Co, Ni, Cu, and Zn as doped-metal species.

The BOP table for the M@Ce_12_O_8_^36+^ cluster models, M = Ce (non-substitution) and M = Ti, V, Cr, Mn, Fe, Co, Ni, Cu, Zn (substitution), is shown in Table 1. The horizontal items show the type of the doped metal, and the vertical items show the valence of the doped metal. The valence of the doped metal was varied from tetravalent (M^4+^) to trivalent (M^3+^) in each 0.05 valent, and each cell was colored by gradient according to the obtained BOP values, blue and red for larger and smaller values, respectively.

As can be seen from Table 1, comparing the BOP values change with the doped metal species and the BOP values with the valence changes, it was found that the BOP value changes are more marked in the difference of the doped metal species. With and without doping the transition metal, the difference shows that the BOP values are large in transition metal-doped ceria than in undoped ceria. This calculation result means that the transition metals doping into ceria would stabilize the ceria crystal structure. If the ceria crystal structure is unstable and its structure completely collapses in the redox reaction, the efficiency of the thermal redox reaction would show less reactivity because it will not have cyclability. On the other hand, if the crystal structure is stable without the collapse of the metal-doped ceria, the reactivity and cyclability of the thermal two-step redox reaction will be enhanced. Moreover, Kodama et al*.* reported that thermal redox reactivity increases with Mn or Fe doped ceria. So, it was suggested that the interatomic bonding in Mn- and Fe-doped ceria becomes stronger than in undoped ceria. It makes the crystal structure stabilization more suitable for thermal redox reactions.

The calculated BOP values were compared with the experimental results of each metal-doped ceria (doped with M = Mn, Fe, Co, Ni). The calculated BOP values of Mn and Fe doped ceria had larger than that of Ni and Cu doped ceria. Therefore, the bond between the doped metal atom and the surrounding oxygen atoms is strong in Mn and Fe doped ceria, which relates to the stoichiometric thermal redox reaction. The bond is weak in Ni and Cu doped ceria, which relates to the nonstoichiometric thermal redox reaction. From this fact, it is understood that when the bonds are strong. The doped ceria crystal does not easily collapse, while when the bonds are weak, the doped ceria crystal easily collapses. The BOP values suggest the contribution of doping to the stabilization of the crystal structure of ceria. Moreover, the BOP values were arranged in M = V, Cr, Mn order for each doped metal species. In the future, we plan to clarify the relationship between structural stability by doping and the oxygen adsorption /desorption reactions through thermodynamic calculations.

Next, the results will be described in the case of doping into the ceria with the *4d* and *5d* transition metal and the lanthanoid metal elements. We report the results of doped ceria with Zr to Cd of the *4d* transition metal element, Hf to Hg of the *5d* transition metal element, and La to Yb of the lanthanoid metal element.

Table 2 shows the BOP values of the M@Ce_12_O_8_^36+^ cluster model, M = Ce (unsubstituted) and M = *4d* and *5d* transition metal elements and the lanthanoid metal elements. The substituted metal elements are arranged according to the periodic table, with the group and the period on the horizontal and the vertical axis, respectively. The valence of the substituted doped metal is set at quadrivalent (M^4+^), and the colors of each cell by gradient according to the obtained BOP value, blue and red for larger and smaller values, respectively.

Table 2 suggested that the metal-doping with the *3d* transition metals stabilizes the ceria crystal structure rather than with the lanthanoid metals. In addition, focusing on the group of doped metal elements, the result of the BOP value was larger in the order of the *3d*, *4d*, and *5d* transition metals. In doping with the *3d*, *4d*, and *5d* transition metal elements, it was easily understood that the stability was changed due to the number of electrons occupied by the outermost orbitals. The bond between the doped metal atom and the oxygen atom (M–O) became more stabilized by doping with the 5, 6, and 7 group metals. On the other hand, doping with the lanthanoid metals, which have the f-orbital in the outer shell, the stability in the ceria crystal structure could not be effective.

Table 2 shows the doping usefulness of the transition metal atoms with *d-*orbitals, and the stability depends on the number of electrons occupied by the *d-*orbitals. In addition, when the *4d* and *5d* transition metals were doped, the crystal structure was more stable than the *3d* transition metals. Therefore, it was considered that the index for the suitable hydrogen production materials was given about the metal-doped ceria material, which has not yet been synthesized.

### Evaluation of p-DOS

The BOP change depending on the doped metal is described in “Evaluation of BOP” Section. Although all of them have the same crystal structure of the fluorite type, the difference in the bond strength could be explained by the magnitude of the interaction between the doped metal and the oxygen atom in the M@Ce_12_O_8_^36+^ cluster model. Here, it is described the features of the doped ceria with the *3d* transition metals, and the relation between the doped metal atoms and the oxygen atoms. The p-DOS results show the *3d* orbital of the transition metal and the *2p* orbital of the first nearest oxygen atom in each M@Ce_12_O_8_^36+^ cluster model. The detailed calculation results are shown in SI. 1 to 12.

**Figure 1:**
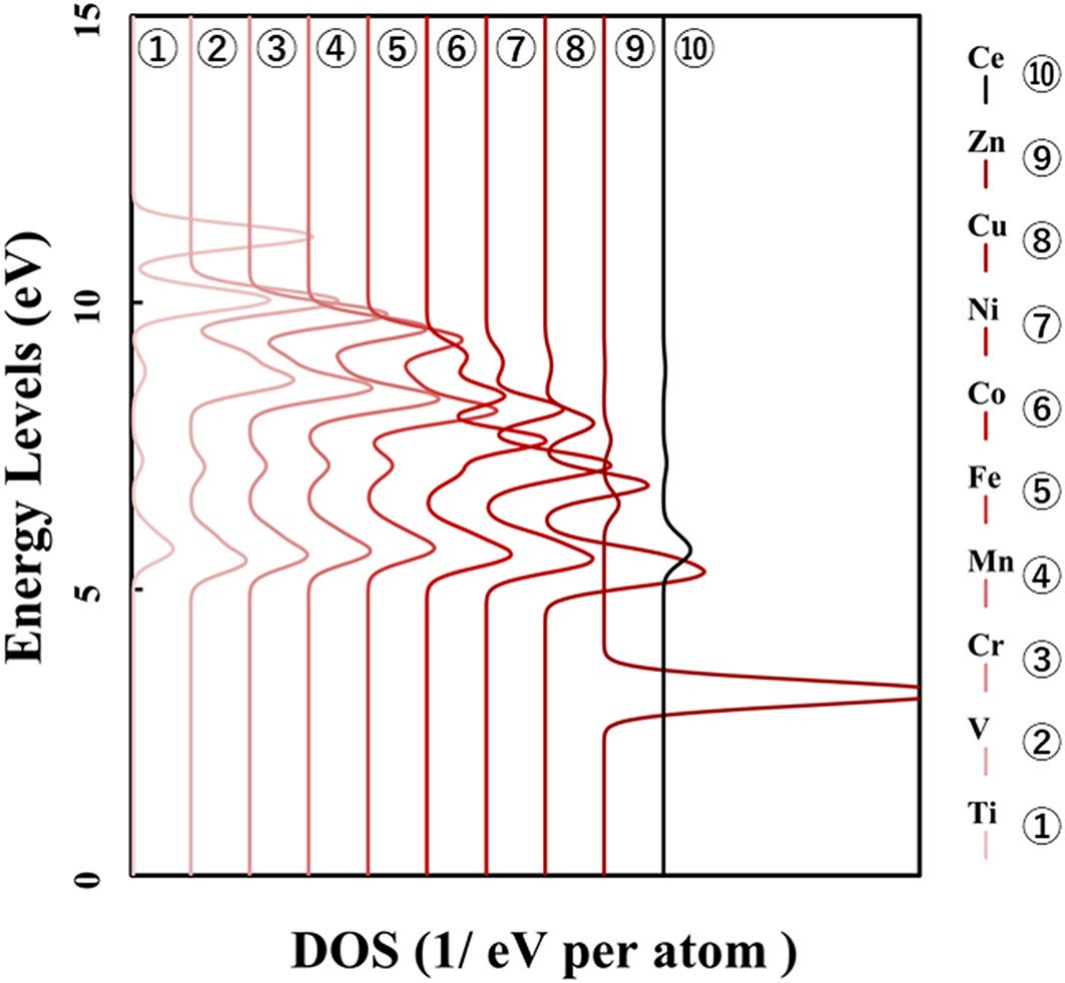
p-DOS for the *3d* orbitals of the doped metal atoms in the M@Ce_12_O_8_^36+^ cluster model, the vacuum level is the reference energy level (M = Ti, V, Cr, Mn, Fe, Co, Ni, Cu, Zn, Ce).

**Figure 2:**
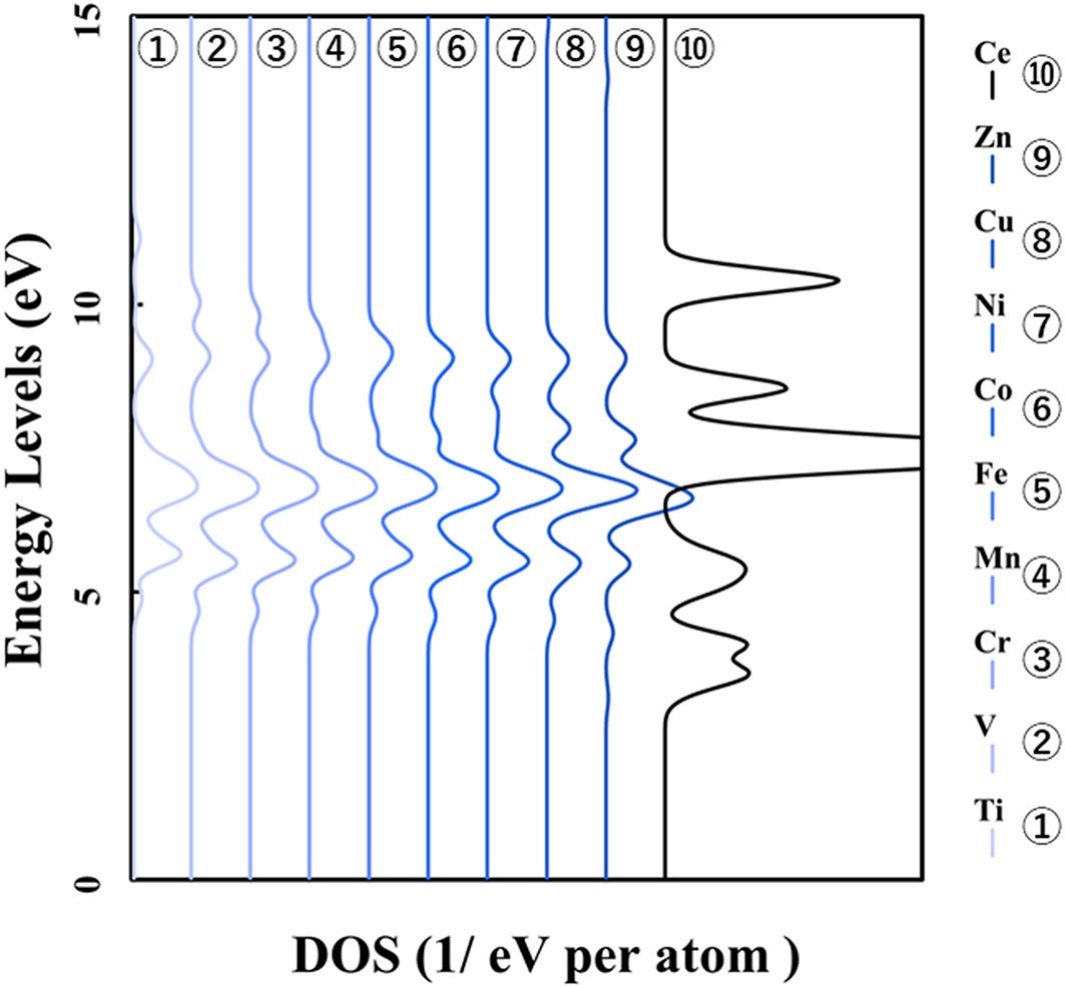
p-DOS for the *2p* orbitals of the oxygen atoms in the M@Ce_12_O_8_^36+^ cluster model, the vacuum level is the reference energy level (M = Ti, V, Cr, Mn, Fe, Co, Ni, Cu, Zn, Ce).

Figure 1 shows the p-DOS in each transition metal's *3d* orbitals, and Fig. 2 shows the p-DOS in the *2p* orbitals of the oxygen atom in each *3d* transition metal-doped M@Ce_12_O_8_^36+^ cluster model (M = Ti, V, Cr, Mn, Fe, Co, Ni, Cu, Zn, Ce). The vacuum level was set as 0 eV as the reference level in both cases. From Fig. 1, doping into ceria significantly changes the electronic state of the *3d* orbitals. The *d-*orbital levels are shifted to lower energy as the atomic number of the doped metal increases. On the other hand, Fig. 2 shows little change in the peak position or peak shift regardless. This result suggested that the effect of doping the transition metal was small on the electronic state of oxygen.

Therefore, it was suggested that the interaction between the oxygen atom and the cerium atoms affected the bond state between the doped metal atom and the oxygen atom (M–O). In other words, the stabilization of the ceria crystal can be achieved by doping transition metal atoms which do not prevent the spatial expansion of electrons on cerium and oxygen atoms. It is because most of the bonds in the ceria crystal are composed of the bonds between the cerium atoms and the oxygen atoms (M–O). The bond strength shown in Tables 1 and 2 results depends on the doped central metal.

**Figure 3:**
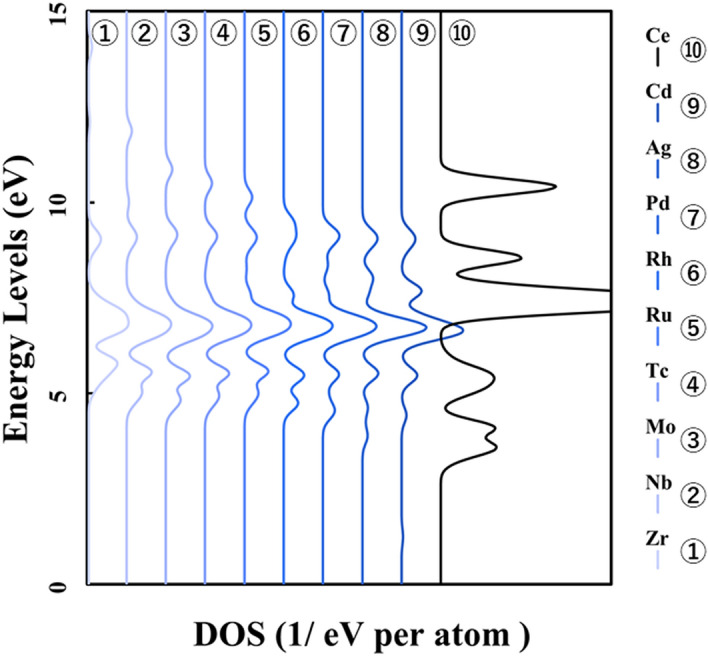
p-DOS for the *2p* orbitals of the oxygen atoms in the M@Ce_12_O_8_^36+^ cluster model, the vacuum level is the reference energy level (M = Zr, Nb, Mo, Tc, Ru, Rh, Pd, Ag, Cd, Ce).

**Figure 4:**
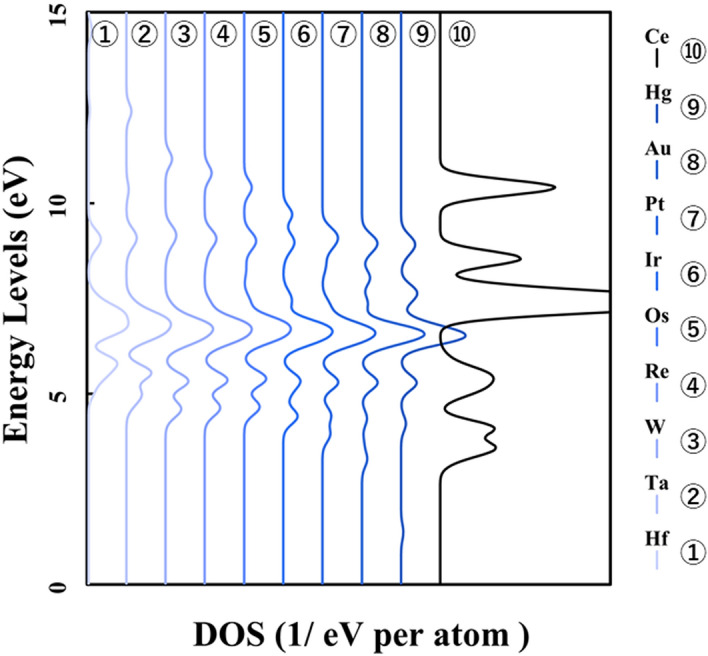
p-DOS for the *2p* orbitals of the oxygen atoms in the M@Ce_12_O_8_^36+^ cluster model, the vacuum level is the reference energy level (M = Hf, Ta, W, Re, Os, Ir, Pt, Au, Hg, Ce).

Next, Figs. 3 and 4 show the result of doping the *4d* and *5d* transition metal into the ceria. The p-DOS in the *2p* orbital of oxygen in Figs. 3 and 4, as in Fig. 2, shows little change in the peak position or peak shift. These results also indicated that there is no significant difference in the behavior of electrons on oxygen atoms and that doped transition metal atoms determine the stability in the ceria crystal structure.

**Figure 5:**
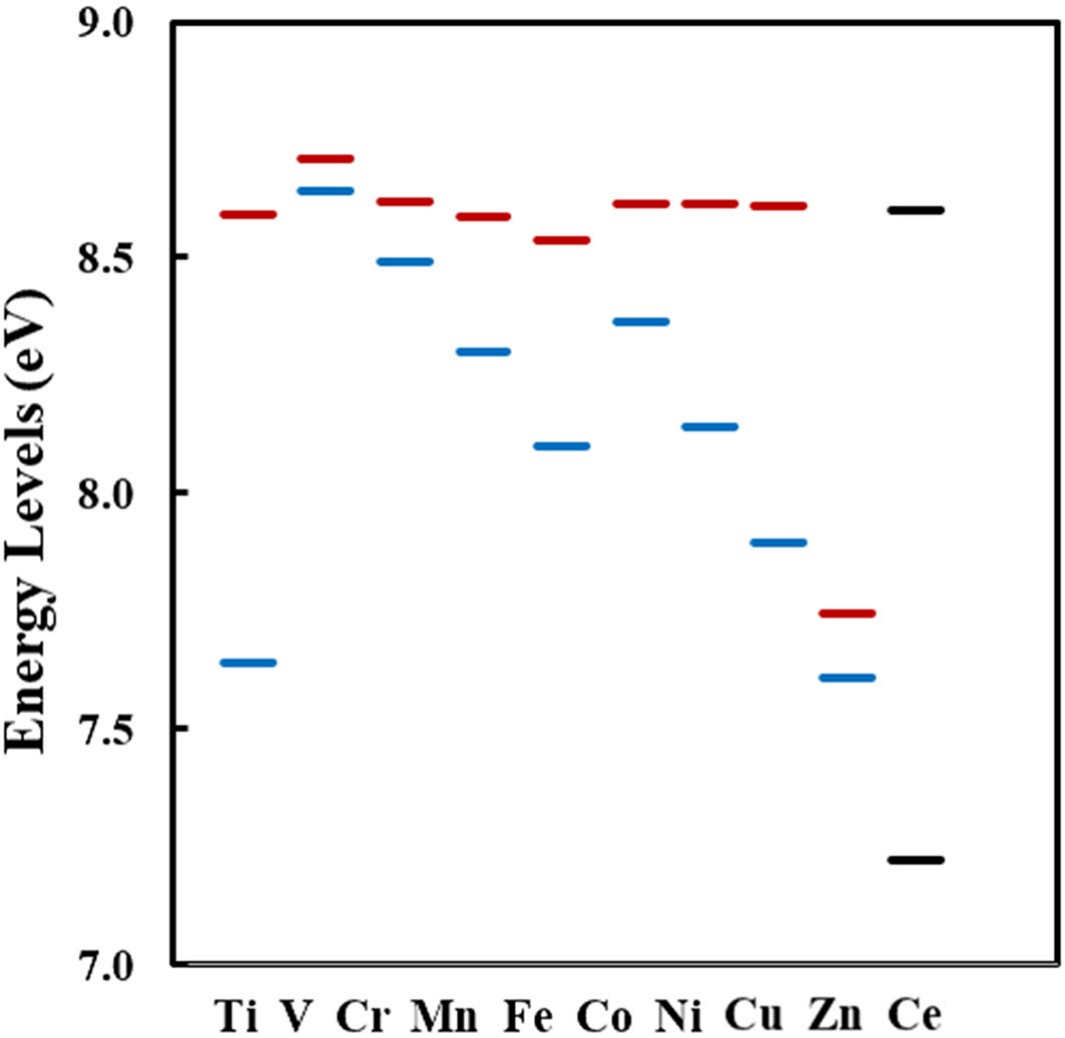
The LUMO (eV), HOMO (eV) level, and the bandgap (eV) in each M@Ce_12_O_8_^36+^ cluster model is based on the reference level as the vacuum level, as described in Table 3. The HOMO level is colored in blue, and the LUMO level is colored in red. In the undoped pure ceria, the HOMO and LUMO levels are colored in black.

It was suggested that the bond strength is formed by the interaction between the doped metal atoms and the oxygen atoms. We confirmed whether the bond strength (the BOP value) between the doped metal atom and oxygen atoms (M–O) is related to the HOMO and LUMO levels and the bandgap in each metal-doped ceria. In Table 3, LUMO and HOMO levels and the bandgap are based on the reference level as the vacuum levels in the M@Ce_12_O_8_^36+^ cluster model. Figure 5 shows the values summarized in Table 3 regarding energy levels on the vertical axis and each doped metal on the horizontal axis. The LUMO and HOMO levels and the bandgap of each M@Ce_12_O_8_^36+^ cluster model are related to the stability in Table 1. The vanadium(V) doped ceria has the lowest bandgap of 0.066 eV, the BOP value of 1.567 (V^4+^) is the largest BOP value among the *3d* metal-doped ceria.

**Table 1:**
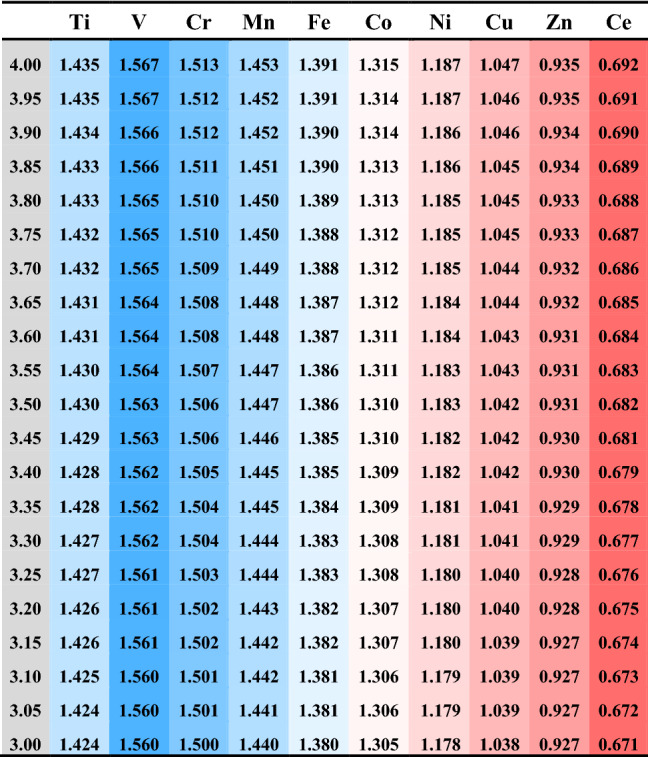
The BOP table for the M@Ce_12_O_8_^36+^ cluster models (M = Ti, V, Cr, Mn, Fe, Co, Ni, Cu, Zn, Ce).

The maximum value (1.967) is set to blue and the minimum value (0.671) to red. Each cell is colored by gradient according to the obtained BOP values, blue for larger and red for smaller values.

**Table 2:**
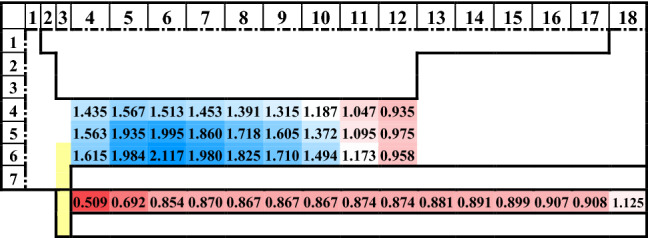
The BOP table for the M@Ce_12_O_8_^36+^ cluster models is arranged according to the period and group of the periodic table.

When the maximum value (2.117) is set to blue and the minimum value (0.509) to red, each cell is colored by gradient according to the obtained BOP value, blue and red for larger and smaller values, respectively.

**Table 3:** The LUMO (eV), HOMO (eV)level, and the bandgap (eV) in each M@Ce_12_O_8_^36+^ cluster model based on the reference level as the vacuum level.

	LUMO	HOMO	Gap
Ti	8.592	7.638	0.954
V	8.708	8.642	0.066
Cr	8.617	8.492	0.125
Mn	8.585	8.300	0.285
Fe	8.537	8.099	0.439
Co	8.615	8.362	0.253
Ni	8.613	8.142	0.472
Cu	8.607	7.893	0.714
Zn	7.744	7.606	0.137
Ce	8.598	7.222	1.376

The bandgap is the largest at 1.376 eV in the undoped ceria, with the BOP value of 0.692 (Ce^4+^). It is the smallest BOP value compared to the *3d* transition metal-doped ceria. However, there is no relationship, such as a smaller bandgap tends to have larger BOP values, or a larger bandgap tends to have smaller BOP values. Next, the HOMO and LUMO levels are explained. The highest HOMO level is 7.638 eV with the V-doped ceria, while the lowest HOMO level is 7.222 eV with the undoped pure ceria. At first, there seemed to be a correlation between the HOMO level and the BOP values. However, there is no correlation between the stability of the ceria crystal structure (BOP values) and the HOMO level, as a similar relationship between the BOP values and the bandgap. Figure 5 shows the LUMO level colored blue and the HOMO level colored red. It indicates no significant difference in the LUMO levels in each metal-doped ceria. Therefore, there is also no relationship between the LUMO level and the BOP value.

The HOMO, LUMO levels, and the bandgap results for *4d* and *5d* transition metals show similar results to the *3d* transition metal-doped ceria. Therefore, there is no relationship between the BOP values and the HOMO, LUMO levels, or bandgap values for each metal-doped. The results of each detailed calculation are summarized in SI 3, 4, 5, and 6.

**Figure 6:**
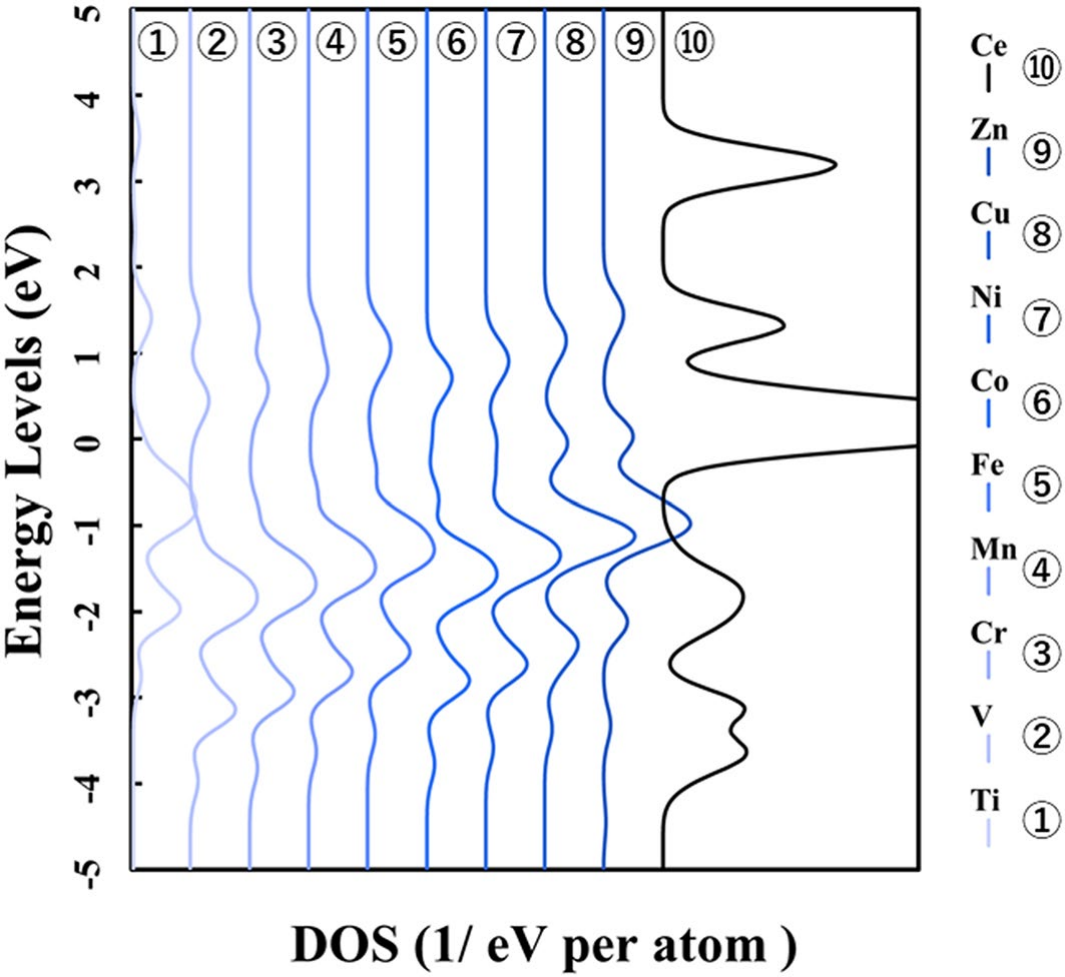
p-DOS for the *2p* orbitals of the oxygen atoms in the M@Ce_12_O_8_^36+^ cluster model, the HOMO level in each M@Ce_12_O_8_^36+^ cluster model is the reference energy level. (M = Ti, V, Cr, Mn, Fe, Co, Ni, Cu, Zn, Ce).

In Fig. 6, p-DOS in the *2p* orbitals of the oxygen atoms are shown in each *3d* transition metal-doped M@Ce_12_O_8_^36+^ cluster model (M = Ti, V, Cr, Mn, Fe, Co, Ni, Cu, Zn, Ce). The HOMO level in each M@Ce_12_O_8_^36+^ cluster model is the reference level. As shown in Fig. 2, there is no difference in the shape of the peak profile, but there is a difference in the peak positions. It was considered that a slight energy shift is one of the effects of metal doping into ceria.

### Evaluation of bonding orbitals and antibonding orbitals

The difference in the bond strength between the doped metal atom and the surrounding oxygen atoms (M–O) greatly affects the stability of the ceria crystal structure. Bonding and antibonding orbitals in molecular orbitals will be described in this section. Interactions between the doped metal atom and the surrounding oxygen atoms in each M@Ce_12_O_8_^36+^ cluster model are explained by interpreting the bonding and antibonding orbitals. The detailed calculation results are shown in SI. 19 to 27.

**Figure 7:**
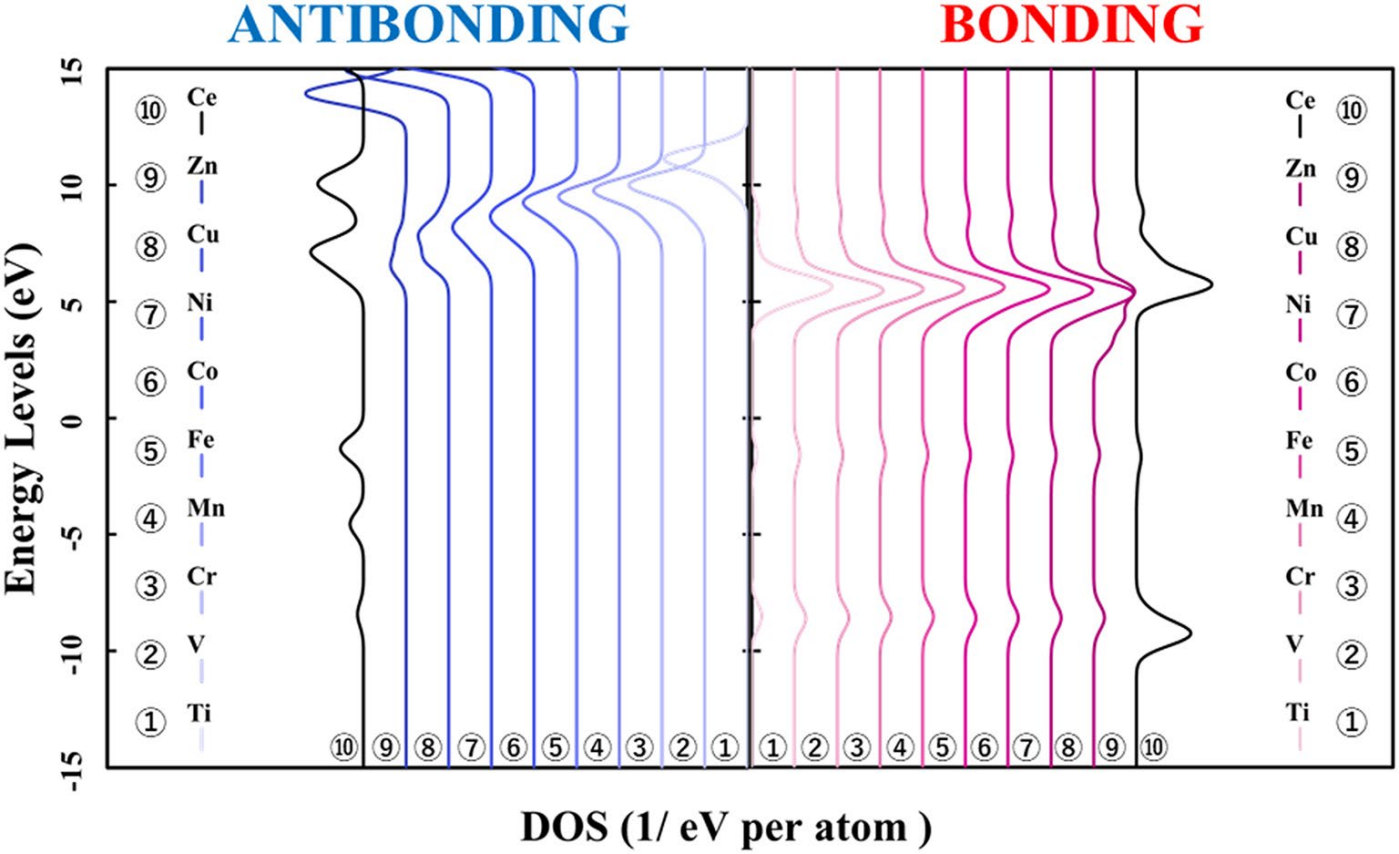
Energy levels of the bond composition between the doped metal and the oxygen atoms are based on the reference level as the vacuum level in the M@Ce_12_O_8_^36+^ cluster model (M = Ti, V, Cr, Mn, Fe, Co, Ni, Cu, Zn, Ce). The bonding and antibonding orbital components are on the right and left sides, respectively.

In Fig. 7, the bonding orbitals between the metal atom and the surrounding oxygen atoms are shown on the right. The antibonding orbitals are shown on the left for each doped metal atom, based on the reference level as the vacuum level in each M@Ce_12_O_8_^36+^ cluster model. From Fig. 7, the peaks of the bonding orbital component are concentrated around at 5–7 eV except for the Zn-doped ceria. It means that there was no difference in the factors which strengthened the bonds even if the kind of the doped metal changed. However, focusing on the antibonding orbital component, the peak of the antibonding orbital component shifts to the lower energy side. So, when the atomic number of the doped metal atom is increased, the orbitals of the antibonding component are more occupied below the HOMO level. Then, it was suggested that more electrons are occupied in the antibonding orbital component, which weakens the bonds between the doped metal atom and the oxygen atoms (M–O).

**Figure 8:**
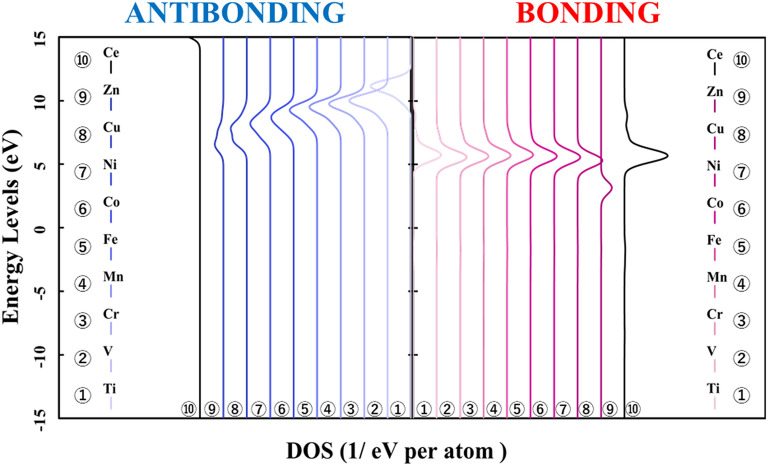
Energy levels of the bond composition between the metal *3d* orbitals and the oxygen *2p* orbitals are based on the reference level as the vacuum level in the M@Ce_12_O_8_^36+^ cluster model (M = Ti, V, Cr, Mn, Fe, Co, Ni, Cu, Zn, Ce). The bond composition between the metal *5d* orbitals and the oxygen *2p* orbitals are in the undoped ceria. The bonding and antibonding orbital components are on the right and left sides, respectively.

**Figure 9:**
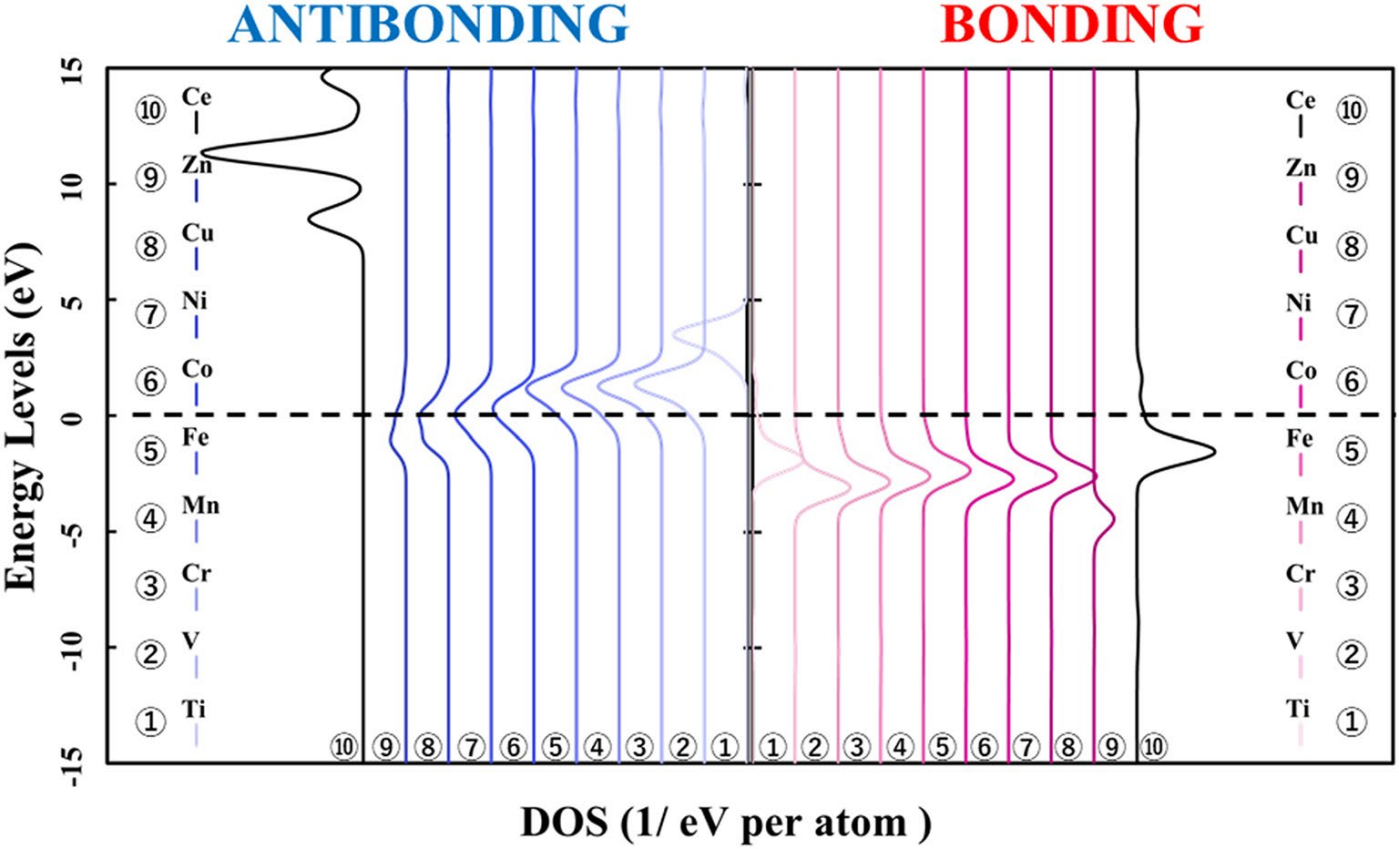
Energy levels of the bond composition between the metal *3d* orbitals and the oxygen *2p* orbitals are based on the reference level as the HOMO level in the M@Ce_12_O_8_^36+^ cluster model (M = Ti, V, Cr, Mn, Fe, Co, Ni, Cu, Zn, Ce). The bond composition between the metal *5d* orbitals and the oxygen *2p* orbitals are in the undoped ceria. The bonding and antibonding orbital components are on the right and left sides, respectively.

Next, it is shown the bonding and antibonding of the *3d* orbitals of the doped metal atom and the *2p* orbitals of the oxygen atoms in Figs. 8 and 9. Then, the energy level in Fig. 8 is based on the reference level as the vacuum level, in Fig. 9 is based on the reference level as the HOMO level in each in the M@Ce_12_O_8_^36+^ cluster model. In Fig. 8, the distribution of the bonding and antibonding orbital components, whose peak does not change in Fig. 7. It indicated that the bonding between the doped metal atom and the oxygen atoms depends on the magnitude of the interaction. The interaction is between the *3d* orbitals and the *2p* orbitals of the oxygen atoms. When the doped metal changes, there is no change in the orbital bonding component between the *3d* orbitals of the doped metal atom and the *2p* orbitals of the oxygen atoms. It was suggested that these factors are reflected in the BOP results shown in Table 1. From the result in Fig. 9, it can be clearly seen that the antibonding orbital component increases below the HOMO level.

Compared Figs. 1 and 2 with Fig. 8, it was understood that the peak shifts of the *3d* orbitals shown in Fig. 1 are as well as the antibonding peaks in Fig. 8. The peak shifts of the p-DOS in the *2p* orbitals in Fig. 2 are as well as the bonding peaks in Fig. 8. This result supports the fact that most of the bonding orbitals are occupied by the *2p* orbitals of the oxygen atoms, while most of the antibonding orbitals are occupied by the *3d* orbitals of the doped metal atom. The antibonding orbital component is mainly occupied by the *3d* orbitals of the doped metal atom, and the energy level also changes depending on the doped metal elements.

**Figure 10:**
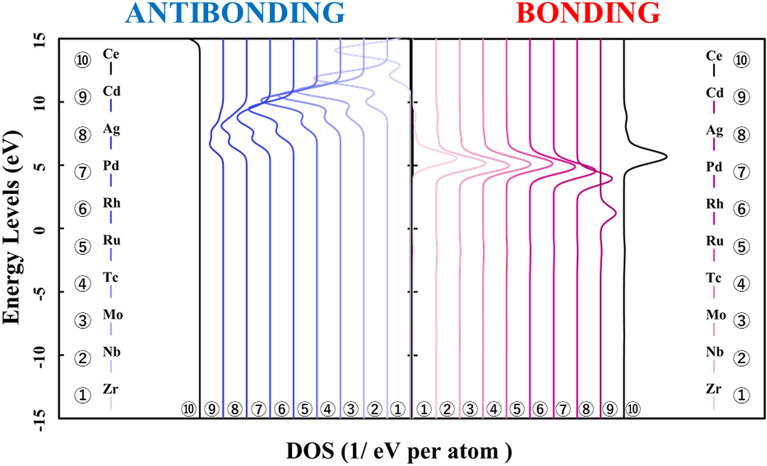
Energy levels of the bond composition between the metal *4d* orbitals and the oxygen *2p* orbitals are based on the reference level as the vacuum level in the M@Ce_12_O_8_^36+^cluster model (M = Zr, Nb, Mo, Tc, Ru, Rh, Pd, Ag, Cd). The bond composition between the metal *5d* orbitals and the oxygen *2p* orbitals are in the undoped ceria. The bonding and antibonding orbital components are on the right and left sides, respectively.

**Figure 11:**
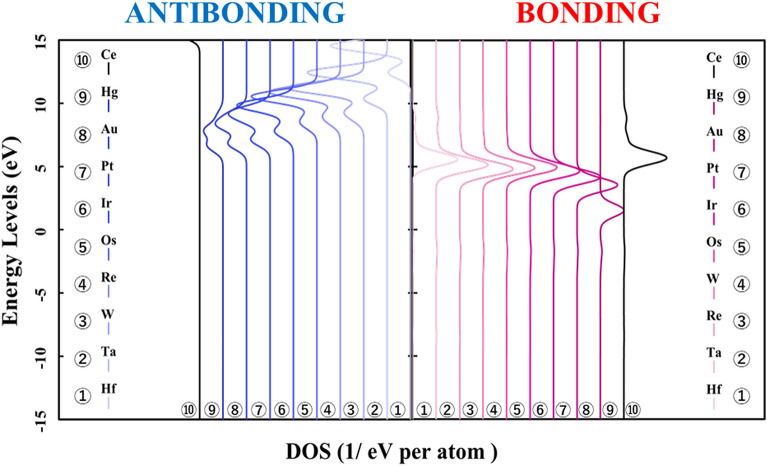
Energy levels of the bond composition between the metal *5d* orbitals and the oxygen *2p* orbitals are based on the reference level as the vacuum level in the M@Ce_12_O_8_^36+^cluster model (M = Hf, Ta, W, Re, Os, Ir, Pt, Au, Hg). The bond composition between the metal *5d* orbitals and the oxygen *2p* orbitals are in the undoped ceria. The bonding and antibonding orbital components are on the right and left sides, respectively.

Here, it is shown that the electronic state of the *2p* orbitals of the oxygen atoms does not change even in the case of not only the *3d* transition metal-doped but also *4d* and *5d* transition metal-doped. The results of the bond composition between the metal *4d*, *5d* orbitals and the oxygen *2p* orbitals show in Figs. 10 and 11 based on the reference level as the HOMO level in each M@Ce_12_O_8_^36+^ cluster model. From these results, the energy level of the antibonding orbital component changes while the bonding orbital component does not change. This tendency is like the case of the *3d* transition metal-doped. Moreover, the magnitude of the bonding orbital is higher in order of the *3d*, *4d*, and *5d* orbitals. Based on the spread of the *d-*orbital, it was suggested that the *5d* transition metal doping stabilizes the cerium oxide crystal structure, as shown in Table 2.

From the calculation results of the bonding and antibonding components between the d orbitals of the doped metal atom and the *2p* orbital of the oxygen atom, it was considered that the oxygen atom is affected by the cerium atoms more than the doped metal atoms in the cerium oxide crystal. As shown in Table 2, the bonds between doped metal atoms and oxygen atoms with *d-*orbitals in their outermost shell orbitals are strong.

**Figure 12:**
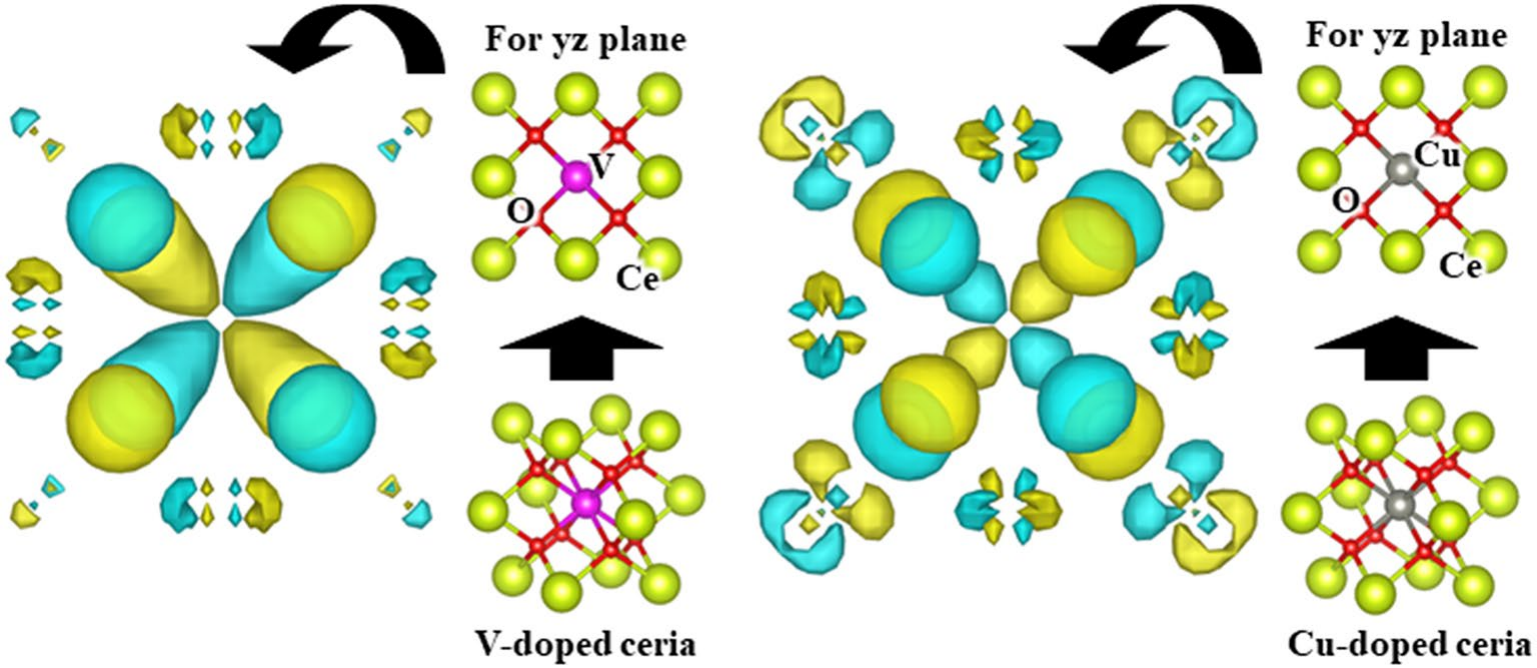
Comparison of the wave functions (visualized by VESTA) of the bonding orbitals: t_2g_ (# 26) in V doped and Cu doped ceria are visualized, the isosurface level is set to 0.025 for both.

As shown in Fig. 12, the wave function spread in the *3d* orbital (#24 t_2g_: d_xy_ orbital) around the Cu metal atom is smaller than that in the case of the V doped. This result suggests that the *d-*orbitals can stabilize the ceria structure in the outermost shell orbitals. An appropriate number of electrons must be occupied in the outermost shell orbitals of the doped metal. Examples are given for the *3d* transition metals doping into the ceria, and the same applies to the case for the *4d* or *5d* transition metals doping into the ceria. Therefore, when a metal classified into groups 5, 6, or 7 is doped, the bonds between the doped metal atom and the oxygen atom become stronger. The repulsion between electrons occupied on the *d-*orbital of the doped metal atom and those occupied on the *2p* orbital of oxygen is not too large, and the electrons occupied on more *d-*orbitals can participate in the bonds between the doped metal atom and the oxygen atoms.

As shown in Table 2, the strong bond is in the order of *3d*, *4d*, and *5d* orbitals. It is attributed to the interaction between the 2p orbitals (the oxygen atom) and the *d-*orbitals (the doped metal atom). Then, more electrons can be shared with the *2p* orbitals of the oxygen atom in the *5d* orbitals. In other words, it is important for stabilizing the ceria crystal structure to dope the metal atoms that do not prevent the spatial spread of cerium atoms and electrons on oxygen atoms in the ceria crystal.

## Conclusion

In the case of the *d-*orbital metal doping, the stability of the interatomic bond can be predicted by the overlap of the wave functions between the *d-*orbitals of the doped metal and the *2p* orbitals of the first nearest oxygen atom. As for the case of transition metal doping in the cerium oxide, the effect of doped metal atoms can be discussed by focusing on the interaction of the outermost orbitals of each atom. By evaluating whether the bonding orbitals between the metal and oxygen atoms are classified as the bonding or antibonding orbitals, it is possible to predict the stability of the crystal structure when the metal atoms are doped into ceria.

A theoretical study was carried out to solve the problem of (i) as mentioned in “Result and discussion” section, considering that the stabilization of crystal structure leads to the improvement of the cyclicity and efficiency of thermal redox reactions. Then, the stabilization of the crystal structure was discussed, focusing on the bonds between the doped metal atom and the oxygen atoms (M–O). Specifically, we concluded that the following factors stabilize the crystal structure when the metal atoms are doped in cerium oxide.

I. The outermost shell orbital of the doped metal atom must be a *d-*orbital.
II. The appropriate number of electrons must be occupied by the *d-*orbitals in the doped metal atom.
III. The principal quantum number of the *d-*orbitals of the doped metal atom must be large.

This study found that the cerium oxide crystal structure is stabilized depending on these factors. In addition, the doped ceria was expected to improve the cyclicity of the two-step thermal reaction and function as a hydrogen generating material whose crystal structure does not collapse during the thermal redox reaction.

## Method

### First principle calculation

This study aims to explain theoretically by using the DV-Xα method. The calculating principle of the Xα method is adopted as a special case of a density functional approach based on the Hohenberg-Korn (HK) theorem^53,54^. The HK state that the ground state energy of a system (assumed to be non-degenerate) is uniquely determined as a function of electron density.

$$\rho \left( r \right) = \sum\limits_{k = 1}^{N} {\left| {\Psi_{k} \left( r \right)} \right|^{2} ,}$$

where N is the number of the sample points. The ${\Psi }_{k}$ is a one-electron wavefunction, which obeys the Hartree–Fock-Slater equation,

$$H\Psi_{k} = E_{k} \Psi_{k} ,$$

Consequently, the one-electron Hamiltonian H is given by

3 $$H = - \frac{1}{2}\nabla_{i}^{2} + V\left( {\varvec{r}} \right) + \int {d\left\lceil b \right\rceil } r^{^{\prime}} \frac{{\rho \left( {r^{^{\prime}} } \right)}}{{\left| {r - r^{^{\prime}} } \right|}} + \frac{{\delta E_{XC} }}{\delta \rho },$$

where V(r) is the Coulomb potential of the nuclei. In the local density approximation (LDA), the last term of Eq. (3) is approximated in the form of $\delta {E}_{XC}$/$\delta \rho$ = *d(*${\rho \varepsilon }_{XC}$*)*/*d* $\rho$. In the Xα method, ${\varepsilon }_{XC}(\rho )$ is the exchange energy of free electron gas multiplied by a constant α:

4 $$\varepsilon_{XC} \left( {\varvec{\rho}} \right) = - 6\alpha \left[ {\frac{3}{4\pi }\rho } \right]^{\frac{1}{3}} ,$$

Setting α = 0.7 usually makes a good approximation. A self-consistent equation is given from the Eqs. (3) and (4). This one-electron approximation is the self-consistent field method (SCF method) proposed by Hartree in 1928 and includes the Hartree–Fock-Slater method proposed by J. C. Slater^52^. The electronic potential proposed by Slater is called “Xα potential,” and the DV-Xα method is another name for the Hartree–Fock-Slater method. The DV-Xα method was developed by D. E. Ellis (Northwestern University) and Hirohiko Adachi (Kyoto University)^47–51^. The molecular wavefunctions were expressed as linear combinations of atomic orbitals (LCAO) obtained by numerically solving the Hartree–Fock–Slater equations in the atom-like potential derived from the molecular potential. Thus, the atomic orbitals as basic functions are automatically optimized for the molecule. The matrix elements in the secular equation,

$$\left( {H - ES} \right)C = 0,$$

are derived from the weighted sum of integrand values at sampling points. The overlap charge densities are partitioned into atomic charges by means of the Mulliken population analysis^55–57^ in the self-consistent charge (SCC) method that was used to approximate the complete self-consistent field. The atomic potentials for generating the basic functions are derived from the spherical average of the molecular charge density around the nuclei, considering of the potential tail of the neighboring atoms. The DV-Xα method has the advantage of numerically evaluating the electronic state of a substance because the secular equation is solved by the numerical integration. Therefore, accurate calculation results can be obtained for the *d-* and *f-*orbitals of the metal ions. Due to the above advantages, the DV-Xα method is used.

### Mulliken population analysis

Considering the linear combination of atomic orbitals (LCAO), the wave functions of a $\ell$ th molecular orbital at an atomic orbital $i$ is shown in below.

$$\phi_{\ell } = \sum C_{i\ell } \chi_{i} ,$$

The Slater's basis function denotes the LCAO coefficients *C* and several atomic orbitals $\chi$. Then, the $q_{ij}^{\ell }$ is the overlap integral $S_{{i{\text{j}}}}$ multiplied by the LCAO coefficient $C_{i\ell } \times C_{j\ell }$.

$$q_{ij}^{\ell } = C_{i\ell } C_{j\ell } \smallint \chi_{i}^{*} \left( {\varvec{r}} \right)\chi_{j} \left( {\varvec{r}} \right)d\user2{r } = C_{i\ell } C_{j\ell } S_{{i{\text{j}}}},$$

**Figure 13:**
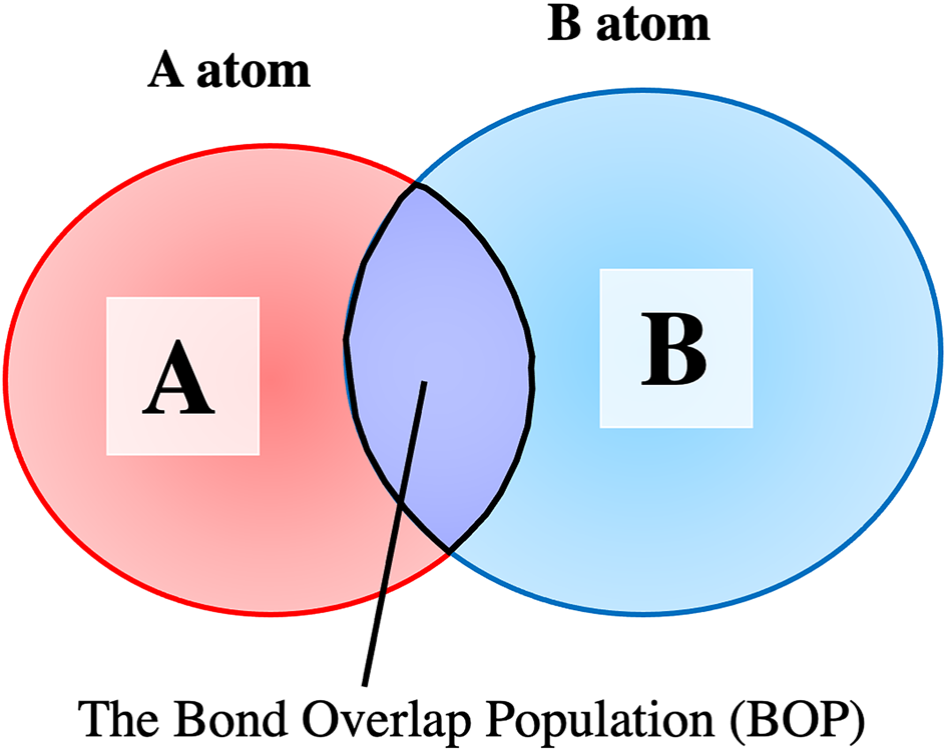
The image of the bond overlap population (BOP) is shown. The color spread is replaced as the wave function in each atom.

These Mulliken analyses allow for an overlap charge of the wavefunctions between atoms. The electrons shared between A and B atom (Fig. 13) are called the effective shared charge (bond overlap population): $Q_{AB}$. The $f_{\ell }$ is the number of occupied molecular orbitals $\ell$ th molecular orbital.

$$Q_{ij} = \mathop \sum \limits_{\ell } f_{\ell } q_{ij}^{\ell } ,$$

$$Q_{AB} = \mathop \sum \limits_{i \in A} \mathop \sum \limits_{j \in B} Q_{ij} ,$$

In this study, the effective shared charge is defined as the bond strength between atoms. From the value of the BOP, we assumed how the crystal structures can be stable.

### Calculation models and terms

We prepared the Ce_13_O_8_^36+^ cluster model to research the electronic states of the cerium atoms and the oxygen atoms. Then, the central cerium atom is substituted for the other metal atom. We tried to see the metal (M) doped effects in Ce_12_MO_8_^36+^ models. The Ce_12_MO_8_^36+^ models mean the central cerium atom changes of the Ce_13_O_8_^36+^ models one after the other. The Ce_12_MO_8_^36+^ models were named M@Ce_12_O_8_^36+^.

We calculated the electronic structures of the M@Ce_12_O_8_^36+^ cluster model composed of 12 cerium (Ce) atoms. It has the quadrivalent per Ce atom and 8 oxygen ions (O^2-^), in which the central atom (M) is substituted with another metal atom (M = *3d*, *4d*, and *5d* transition metals, and the lanthanoid metals) in each calculation model. In other words, we substituted the central M atom with the *3d* (M = Ti, V, Cr, Mn, Fe, Co, Ni, Cu, Zn), *4d* (M = Zr, Nb, Mo, Tc, Ru, Rh, Pd, Ag, Cd), and *5d* transition metal (M = Hf, Ta, W, Re, Os, Ir, Au, Hg), or the lanthanoid metal (M = La, Ce, Pr, Nd, Pm, Sm, Eu, Gd, Tb, Dy, Ho, Er, Ho, Tm, Yb, Lu). Then, M = Ce doped model (Ce@Ce_12_O_8_^36+^  = Ce_13_O_8_^36+^) means that the cluster model is the pure cerium oxide with no substitution. The reported experimental data determined the absolute coordinates of the calculated cluster model. The M@Ce_12_O_8_^36+^ cluster model has a high symmetry of the fluorite type structure (Fm-3 m), and the lattice constant of 5.411 Å. It has the central metal atom(M), the 8 oxygen atoms around the central atom, and 12 cerium atoms located outside each oxygen atom.

The calculations are performed self-consistently until the difference in orbital populations between the initial and final states of the iteration by means of the DV-Xα molecular orbital method is less than 0.0005 electrons. The calculations were also performed considering the Madelung potential in each cluster model (Supplementary Information).

## Supplementary Information

Supplementary Information.

## Data Availability

All data generated or analysed during this study are included in this published article.
