# Supplementary material for: Transition metals doped effects for the crystal stabilization of the cerium oxides with the first principle calculation
Source: Sci Rep. 2022 Jun 16;12:10103. doi: 10.1038/s41598-022-14180-3 (PMC9203553; doi:10.1038/s41598-022-14180-3)
Supplement: Supplementary file 1 — Supplementary Information. [file 41598_2022_14180_MOESM1_ESM.docx]

The lists of calculation results in each M@Ce_12_O_8_^36+^ cluster model used for the one-electron calculation (DV-Xα molecular orbital method).

Figs. SI 1 to S6. Evaluation of p-DOS, the vacuum level is the reference energy level.

The change in electronic states due to the doped metal changes

in each M@Ce_12_O_8_^36+^ cluster model.

Figs. SI 7 to S12. Evaluation of p-DOS, the HOMO level in each M@Ce_12_O_8_^36+^ cluster model is the reference energy level.

Figs. SI 13 to S18. Evaluation of the HOMO, LUMO levels and bandgaps.

Figs. SI 13 to S18. Evaluation of the HOMO, LUMO levels and bandgaps.

Figs. SI 19 to S24. Evaluation of the bonding orbital component and antibonding orbital component, the vacuum level is the reference energy level.

Figs. SI 25 to S27. Evaluation of the bonding orbital component and antibonding orbital component, the HOMO level in each M@Ce_12_O_8_^36+^ cluster model is the reference energy level.

Figs. SI 28 to S47. Evaluation of p-DOS, the vacuum level is the reference energy level.

The change in electronic states due to valence changes in each M@Ce_12_O_8_^36+^ cluster model

(M = Ti, V, Cr, Mn, Fe, Co, Ni, Cu, Zn, Ce).


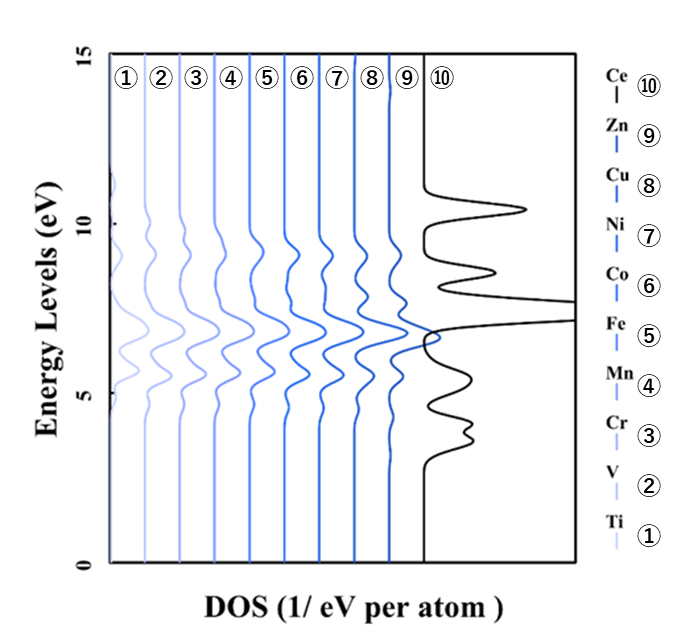


Fig. SI 1. (= Fig. 2) p-DOS for the 2p orbitals of the oxygen atoms in the M@Ce_12_O_8_^36+^ cluster model, the vacuum level is the reference energy level (M = Ti, V, Cr, Mn, Fe, Co, Ni, Cu, Zn, Ce).


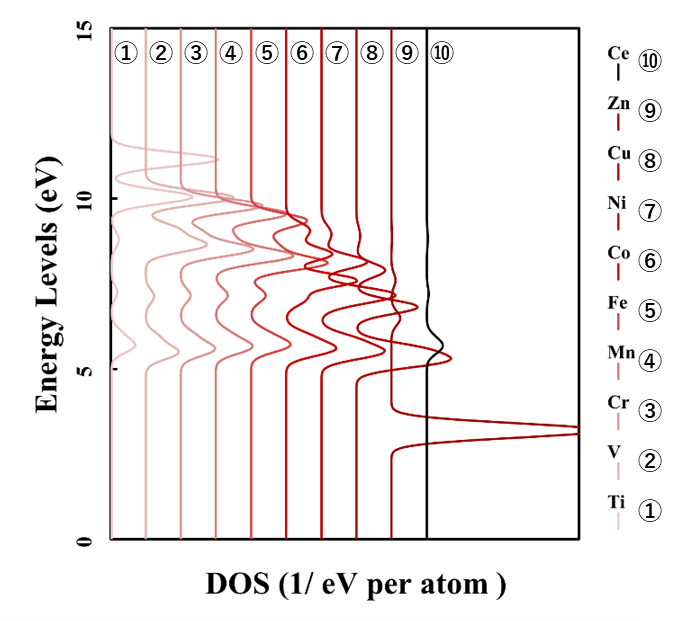


Fig. SI 2. (= Fig. 1) p-DOS for the 3d orbitals of the doped metal atom in the M@Ce_12_O_8_^36+^ cluster model, the vacuum level is the reference energy level (M = Ti, V, Cr, Mn, Fe, Co, Ni, Cu, Zn, Ce).


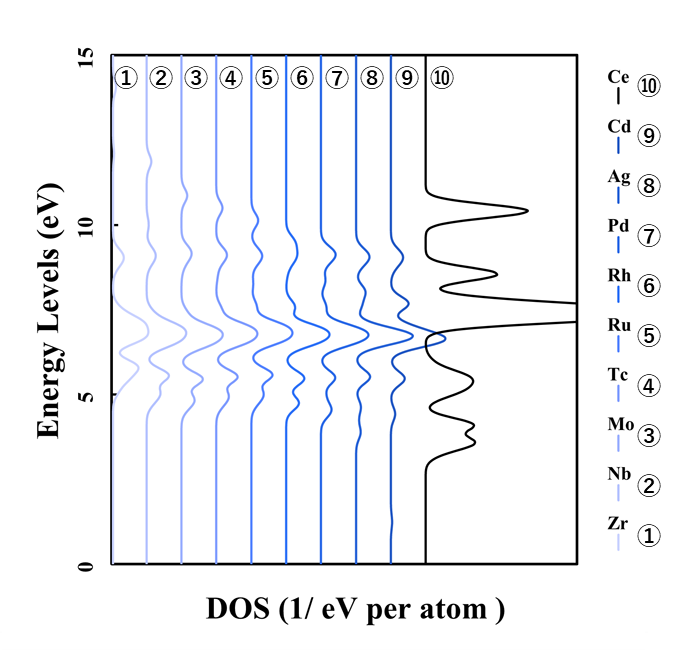


Fig. SI 3. (= Fig. 3) p-DOS for the 2p orbitals of the oxygen atoms in the M@Ce_12_O_8_^36+^ cluster model, the vacuum level is the reference energy level (M = Zr, Nb, Mo, Tc, Ru, Rh, Pd, Ag, Cd, Ce).


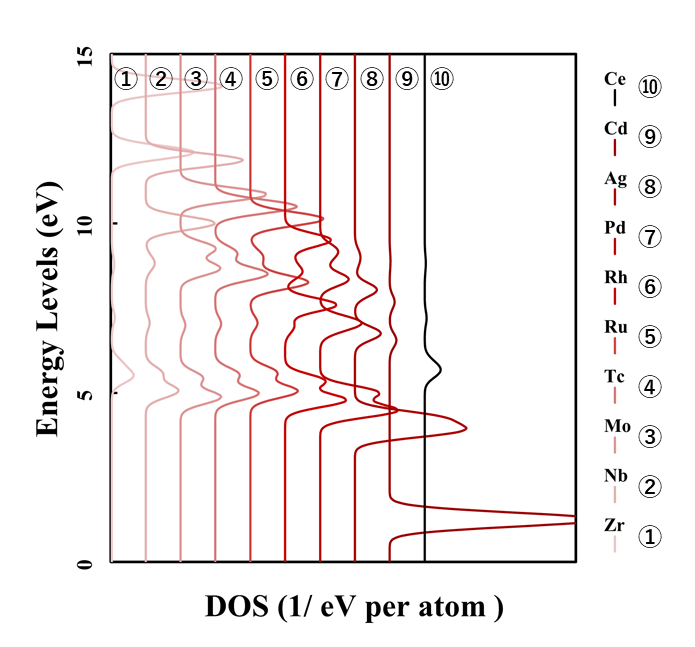


Fig. SI 4. p-DOS for the 4d orbitals of the doped metal atom in the M@Ce_12_O_8_^36+^ cluster model, the vacuum level is the reference energy level (M = Zr, Nb, Mo, Tc, Ru, Rh, Pd, Ag, Cd, Ce).


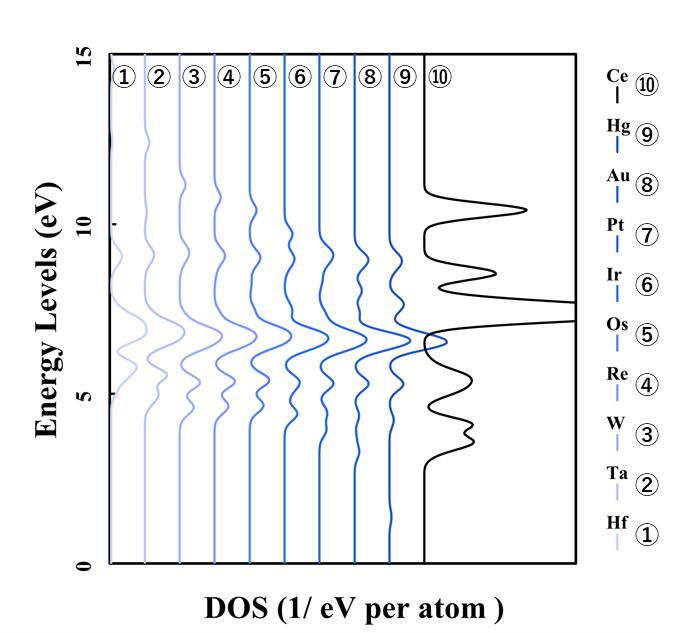


Fig. SI 5. (= Fig. 4.) p-DOS for the 2p orbitals of the oxygen atoms in the M@Ce_12_O_8_^36+^ cluster model, the vacuum level is the reference energy level (M = Hf, Ta, W, Re, Os, Ir, Pt, Au, Hg, Ce).


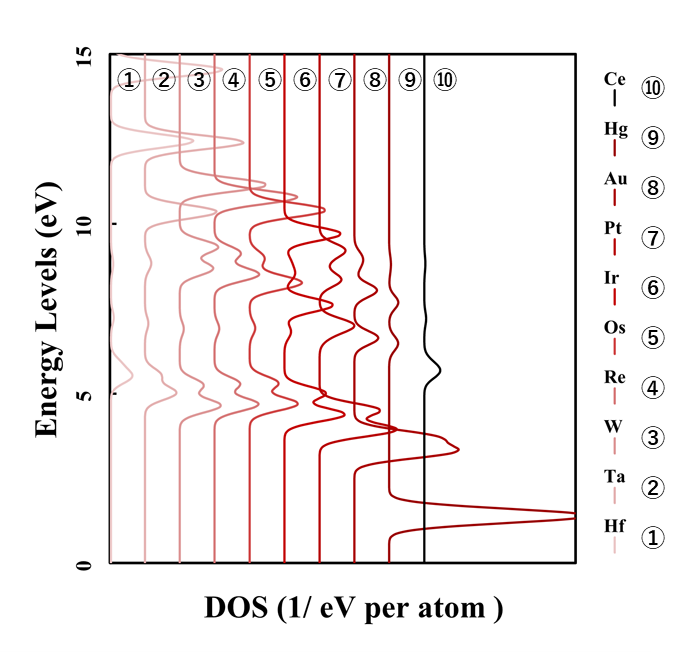


Fig. SI 6. p-DOS for the 5d orbitals of the doped metal atom in the M@Ce_12_O_8_^36+^ cluster model, the vacuum level is the reference energy level (M = Hf, Ta, W, Re, Os, Ir, Pt, Au, Hg, Ce).


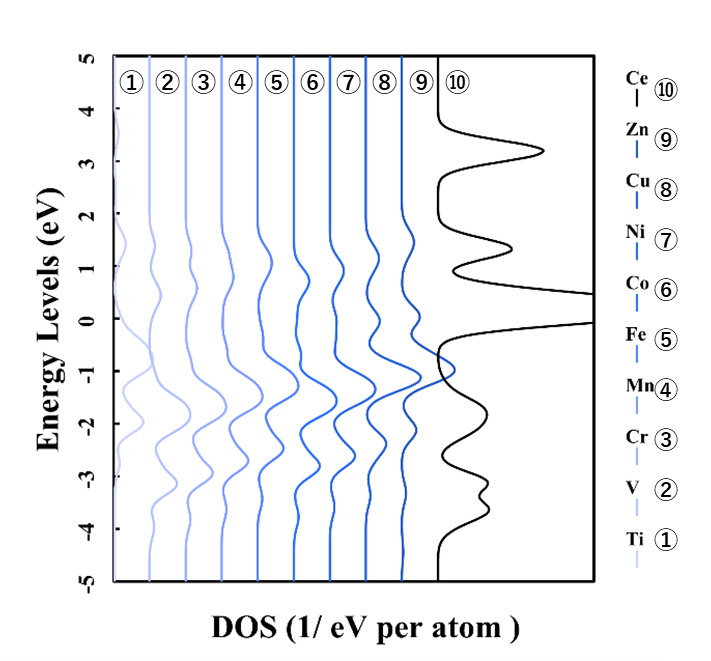


Fig. SI 7. p-DOS for the 2p orbitals of the oxygen atoms in the M@Ce_12_O_8_^36+^ cluster model, the HOMO level in each cluster model is the reference energy level (M = Ti, V, Cr, Mn, Fe, Co, Ni, Cu, Zn, Ce).


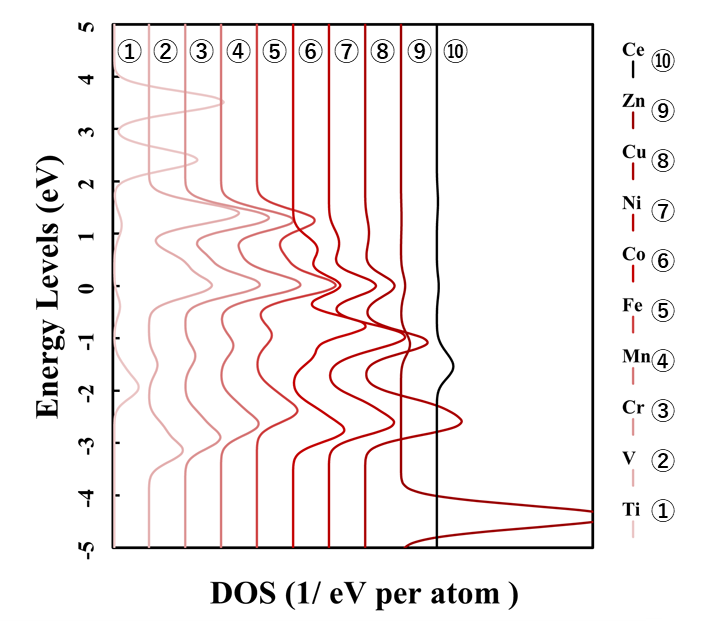


Fig. SI 8. p-DOS for the 3d orbitals of the doped metal atom in the M@Ce_12_O_8_^36+^ cluster model, the HOMO level in each cluster model is the reference energy level (M = Ti, V, Cr, Mn, Fe, Co, Ni, Cu, Zn, Ce).


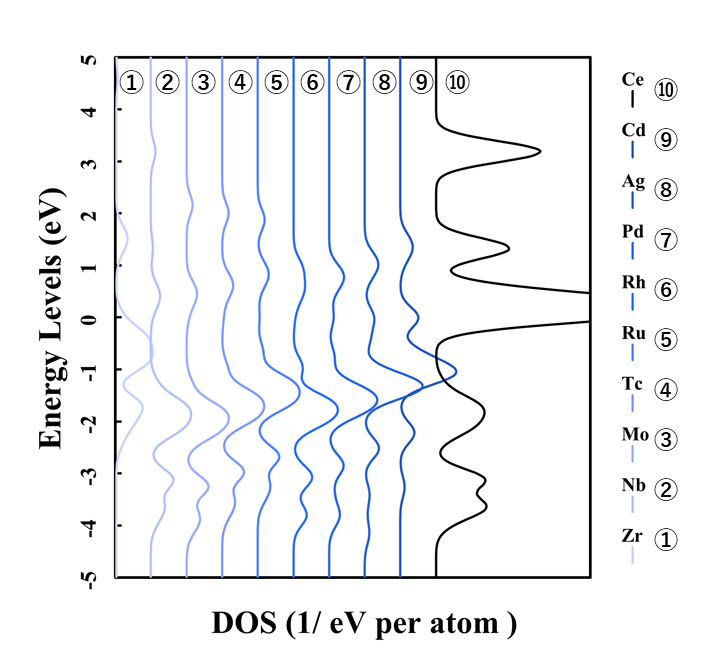


Fig. SI 9. p-DOS for the 2p orbitals of the oxygen atoms in the M@Ce_12_O_8_^36+^ cluster model, the HOMO level in each cluster model is the reference energy level (M = Zr, Nb, Mo, Tc, Ru, Rh, Pd, Ag, Cd, Ce).


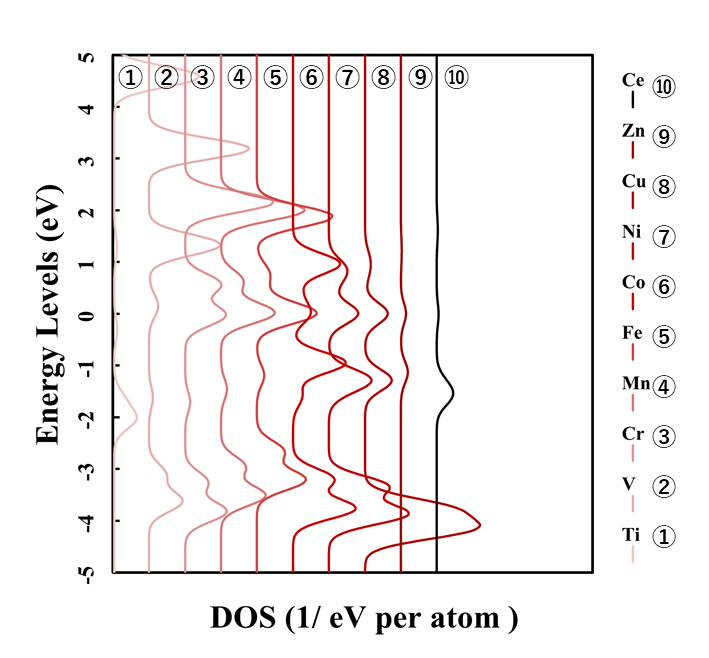


Fig. SI 10. p-DOS for the 4d orbitals of the doped metal atom in the M@Ce_12_O_8_^36+^ cluster model, the HOMO level in each cluster model is the reference energy level (M = Zr, Nb, Mo, Tc, Ru, Rh, Pd, Ag, Cd, Ce).


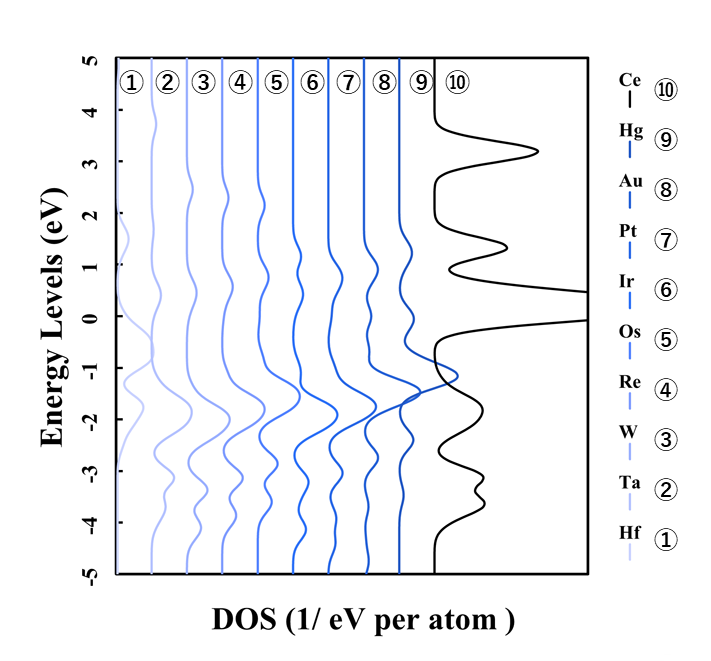


Fig. SI 11. p-DOS for the 2p orbitals of the oxygen atoms in the M@Ce_12_O_8_^36+^ cluster model, the HOMO level in each cluster model is the reference energy level (M = Hf, Ta, W, Re, Os, Ir, Pt, Au, Hg, Ce).


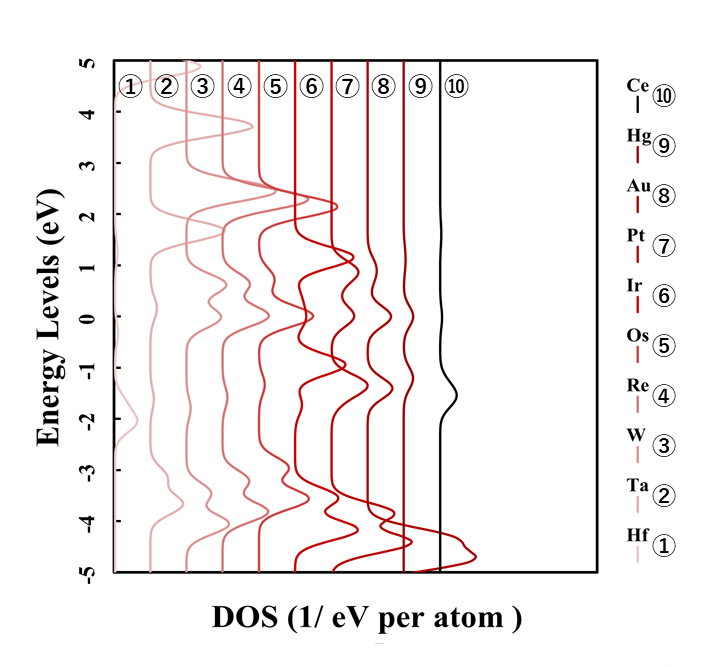


Fig. SI 12. p-DOS for the 5d orbitals of the doped metal atom in the M@Ce_12_O_8_^36+^ cluster model, the HOMO level in each cluster model is the reference energy level (M = Hf, Ta, W, Re, Os, Ir, Pt, Au, Hg, Ce).


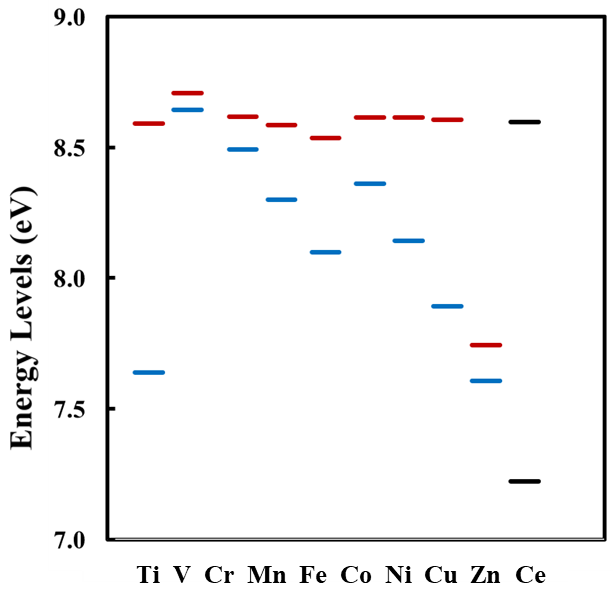


Fig. SI 13. (= Fig. 5) The LUMO, HOMO level, and the bandgap in each M@Ce_12_O_8_^36+^ cluster model with the vacuum level. The HOMO, LUMO level is colored in blue and red, respectively. In the undoped pure ceria, the energy levels are colored in black (M = Ti, V, Cr, Mn, Fe, Co, Ni, Cu, Zn, Ce).

Table SI 14. (= Table 3)　The value of the LUMO, HOMO level, and the bandgap in each M@Ce_12_O_8_^36+^ cluster model with the vacuum level (M = Ti, V, Cr, Mn, Fe, Co, Ni, Cu, Zn, Ce).


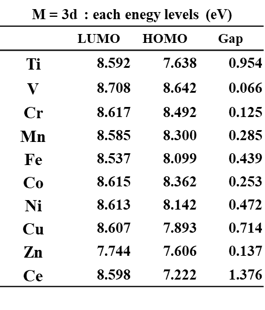


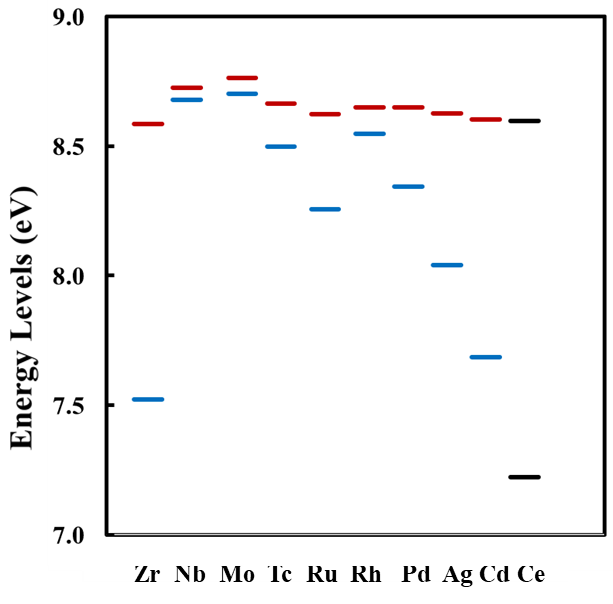


Fig. SI 15. The LUMO, HOMO level, and the bandgap in each M@Ce_12_O_8_^36+^ cluster model with the vacuum level. The HOMO, LUMO level is colored in blue and red, respectively. In the undoped pure ceria, the energy levels are colored in black (M = Zr, Nb, Mo, Tc, Ru, Rh, Pd, Ag, Cd, Ce).

Table SI 16. The value of the LUMO, HOMO level, and the bandgap in each M@Ce_12_O_8_^36+^ cluster model with the vacuum level (M = Zr, Nb, Mo, Tc, Ru, Rh, Pd, Ag, Cd, Ce).


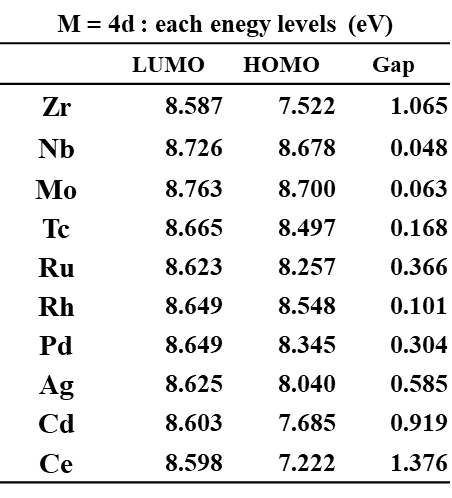


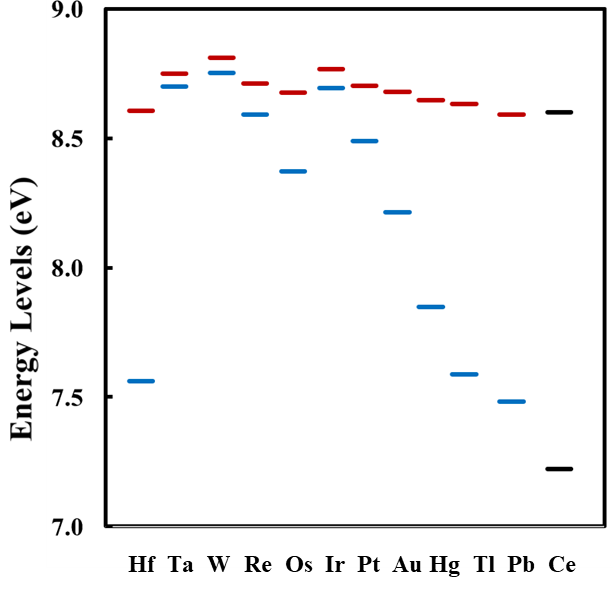


Fig. SI 17. The LUMO, HOMO level, and the bandgap in each M@Ce_12_O_8_^36+^ cluster model with the vacuum level. The HOMO, LUMO level is colored in blue and red, respectively. In the undoped pure ceria, the energy levels are colored in black (M = Hf, Ta, W, Re, Os, Ir, Pt, Au, Hg, Tl, Pb Ce).

Table SI 18. The value of the LUMO, HOMO level, and the bandgap in each M@Ce_12_O_8_^36+^ cluster model with the vacuum level (M = Hf, Ta, W, Re, Os, Ir, Pt, Au, Hg, Tl, Pb Ce).


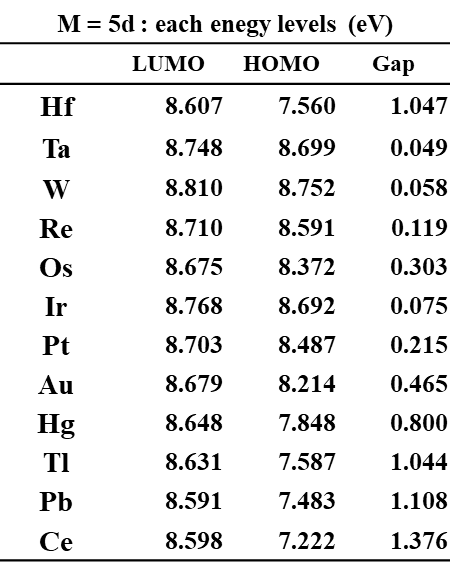


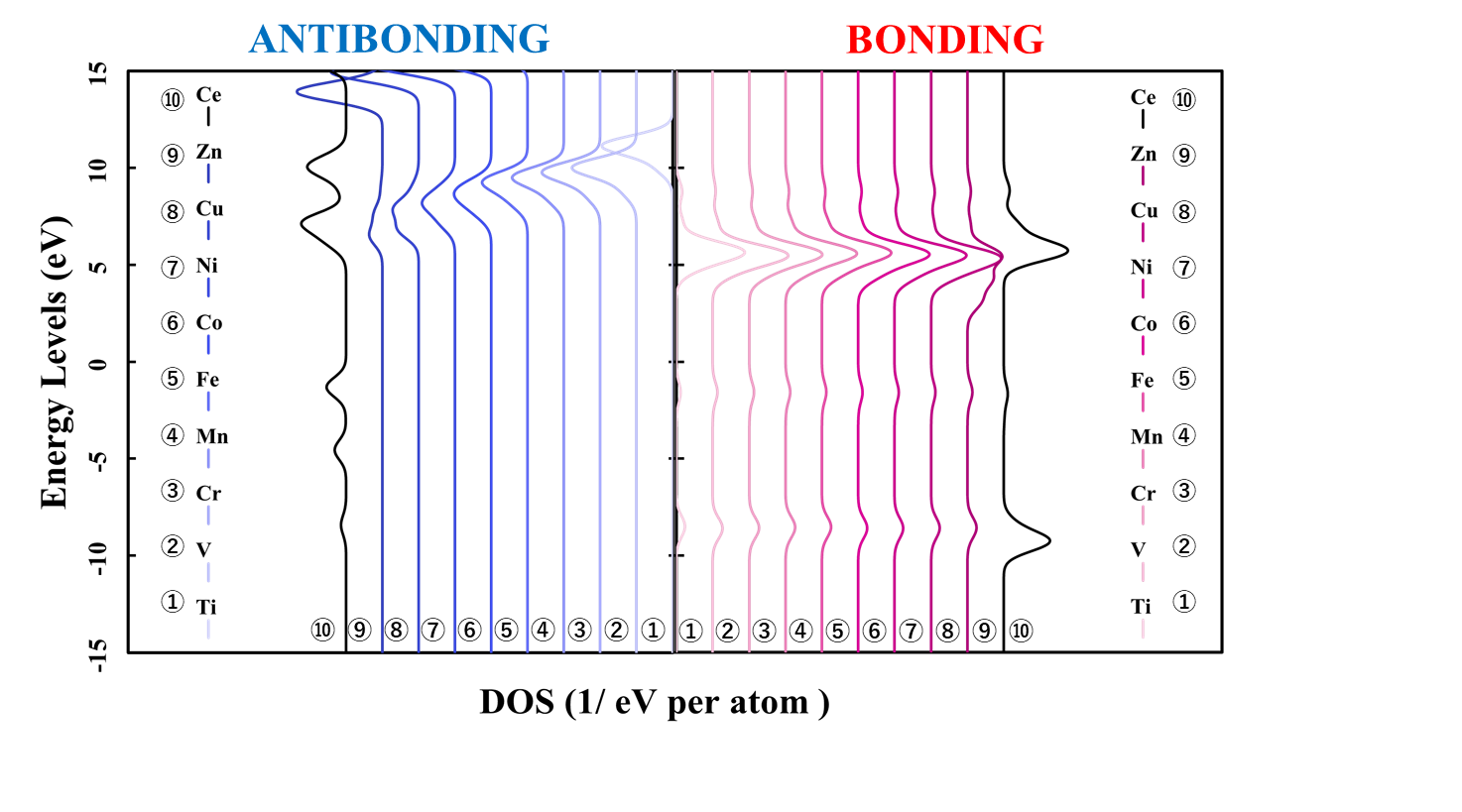


Fig. SI 19. (= Fig. 7) Energy levels of the bond composition between the doped metal and the oxygen atoms with the vacuum level in the M@Ce_12_O_8_^36+^cluster model (M = Ti, V, Cr, Mn, Fe, Co, Ni, Cu, Zn, Ce). The bonding and antibonding orbital components are on the right and left side, respectively.


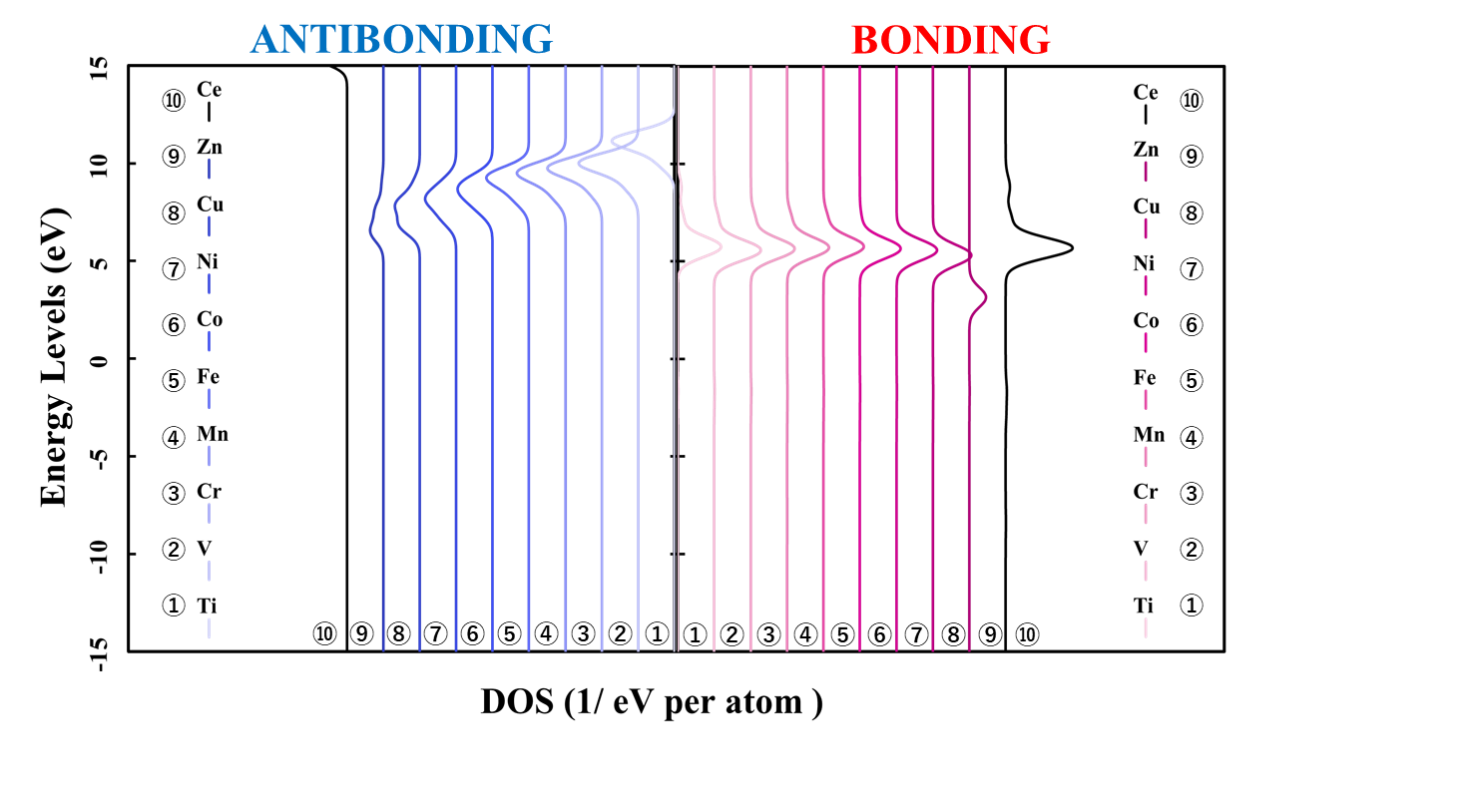


Fig. SI 20. (= Fig. 8) Energy levels of the bond composition between the metal 3d orbitals and the oxygen 2p orbitals in the vacuum level in the M@Ce_12_O_8_^36+^cluster model (M = Ti, V, Cr, Mn, Fe, Co, Ni, Cu, Zn, Ce). In the undoped ceria, the bond composition between the metal 5d orbitals and the oxygen 2p orbitals. The bonding and antibonding orbital components are on the right and left side, respectively.


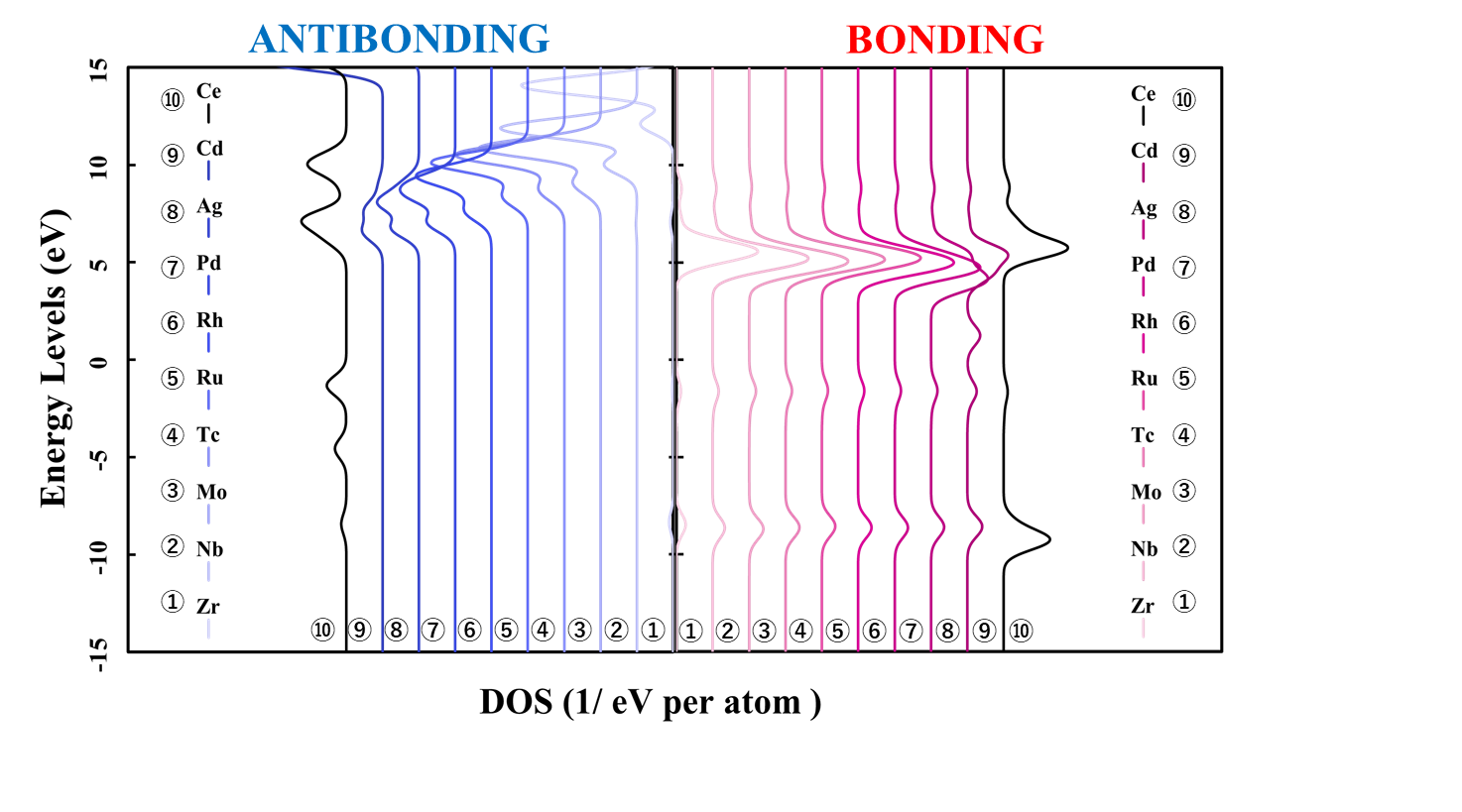


Fig. SI 21. Energy levels of the bond composition between the doped metal and the oxygen atoms with the vacuum level in the M@Ce_12_O_8_^36+^cluster model (M = Zr, Nb, Mo, Tc, Ru, Rh, Pd, Ag, Cd, Ce). The bonding and antibonding orbital components are on the right and left side, respectively.


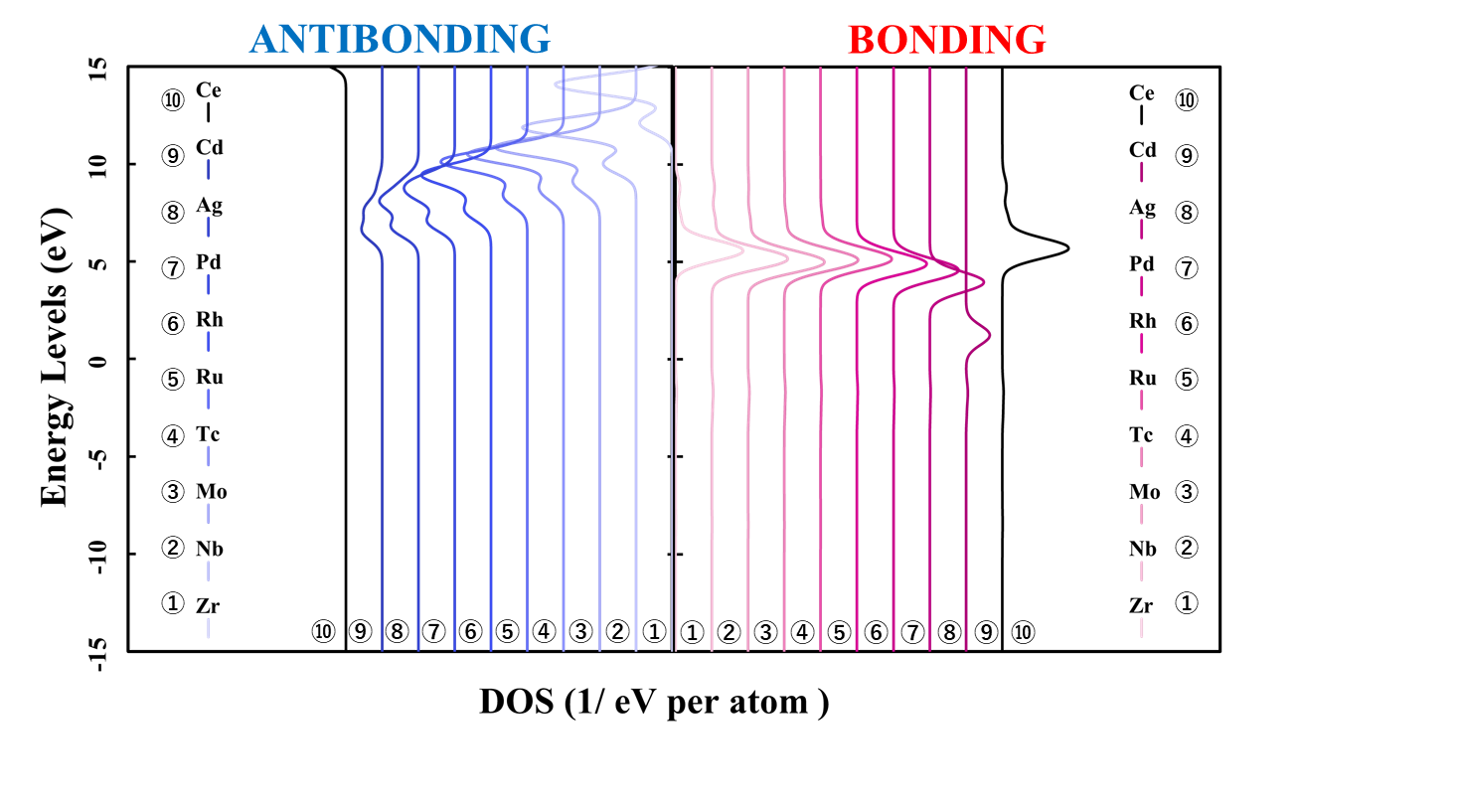


Fig. SI 22. (= Fig. 10) Energy levels of the bond composition between the metal 4d orbitals and the oxygen 2p orbitals in the vacuum level in the M@Ce_12_O_8_^36+^cluster model (M = Zr, Nb, Mo, Tc, Ru, Rh, Pd, Ag, Cd, Ce). In the undoped ceria, the bond composition between the metal 5d orbitals and the oxygen 2p orbitals. The bonding and antibonding orbital components are on the right and left side, respectively.


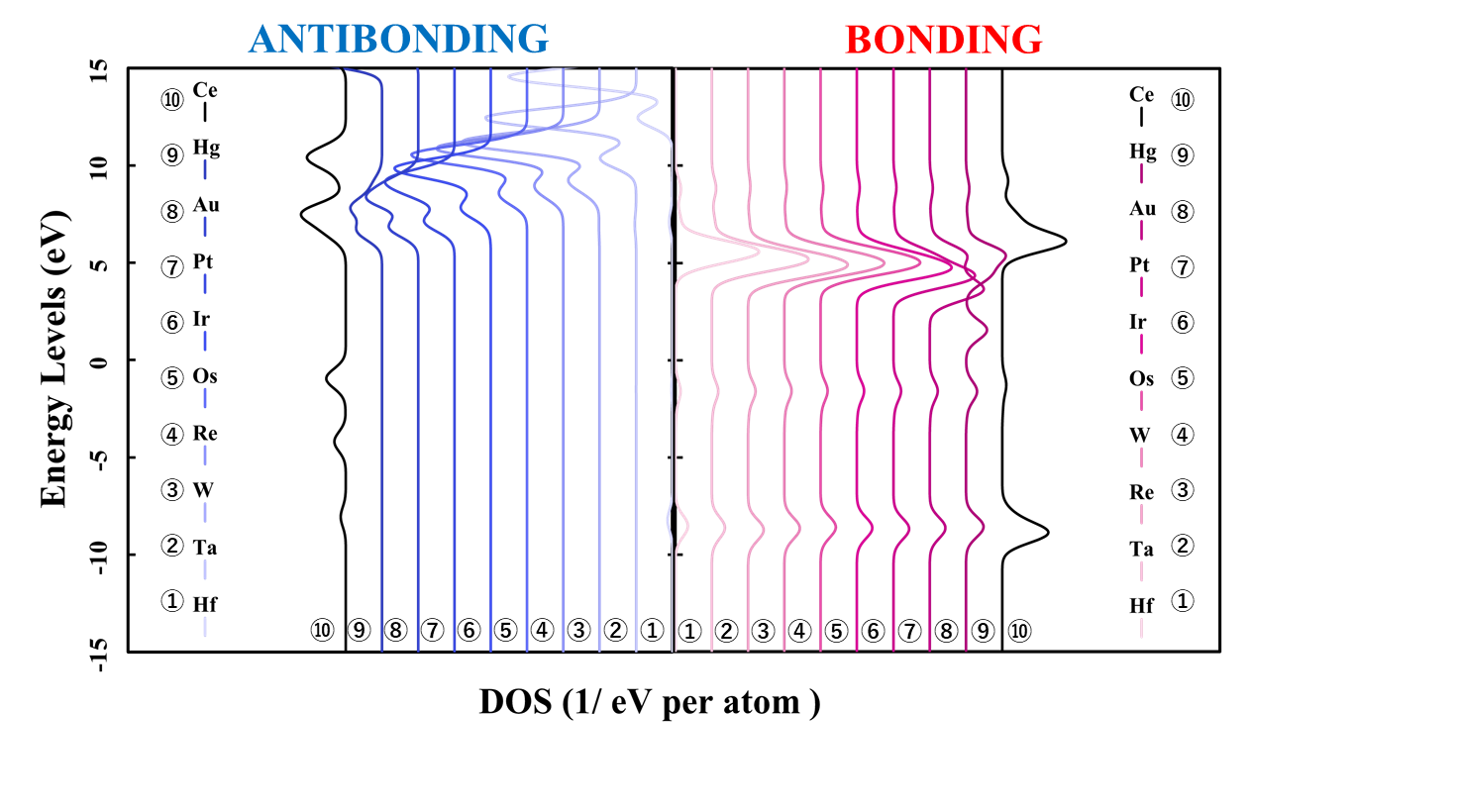


Fig. SI 23. Energy levels of the bond composition between the doped metal and the oxygen atoms with the vacuum level in the M@Ce_12_O_8_^36+^cluster model (M = Hf, Ta, W, Re, Os, Ir, Pt, Au, Hg, Ce). The bonding and antibonding orbital components are on the right and left side, respectively.


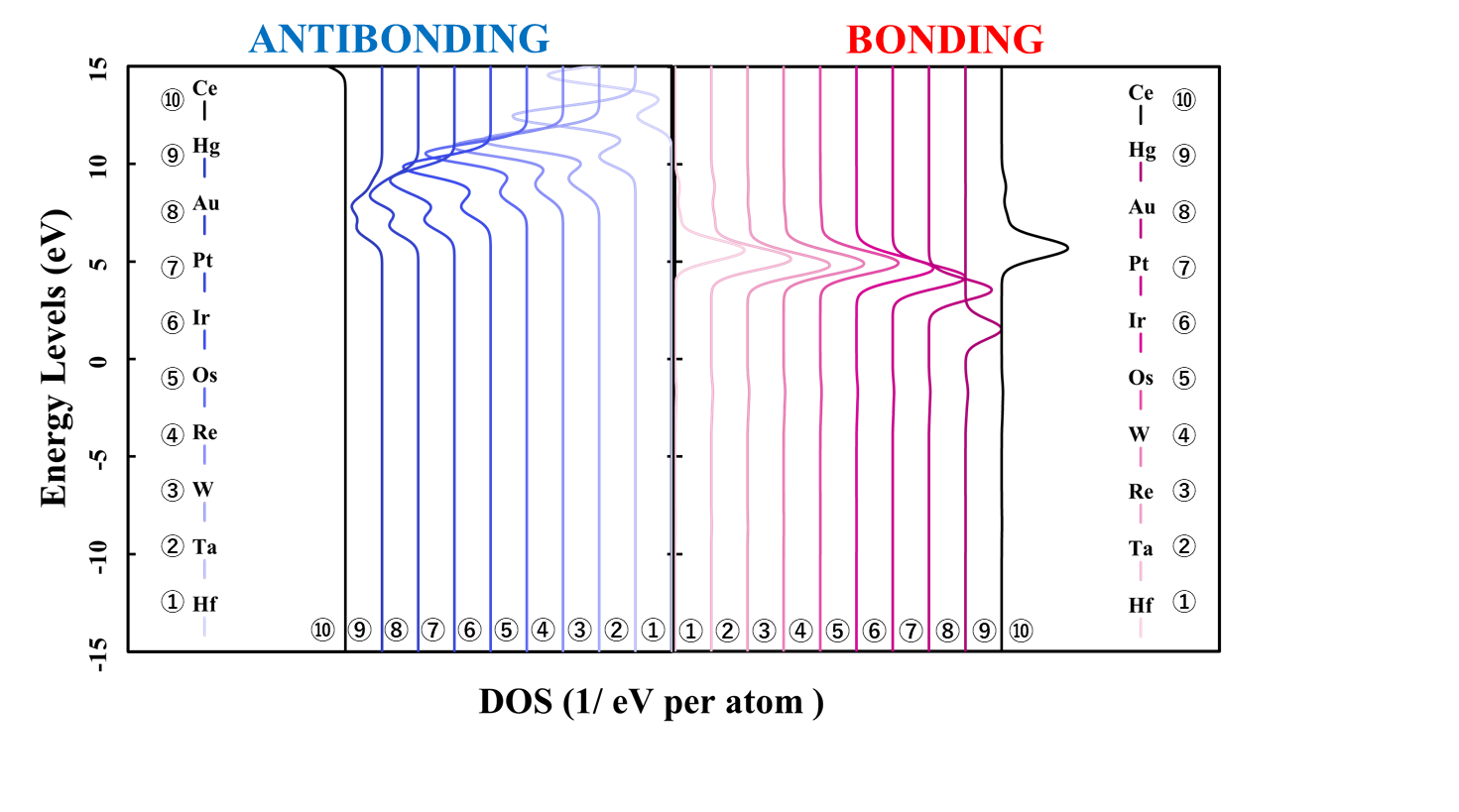


Fig. SI 24. (= Fig. 11) Energy levels of the bond composition between the metal 5d orbitals and the oxygen 2p orbitals in the vacuum level in the M@Ce_12_O_8_^36+^cluster model (M = Hf, Ta, W, Re, Os, Ir, Pt, Au, Hg, Ce). In the undoped ceria, the bond composition between the metal 5d orbitals and the oxygen 2p orbitals. The bonding and antibonding orbital components are on the right and left side, respectively.


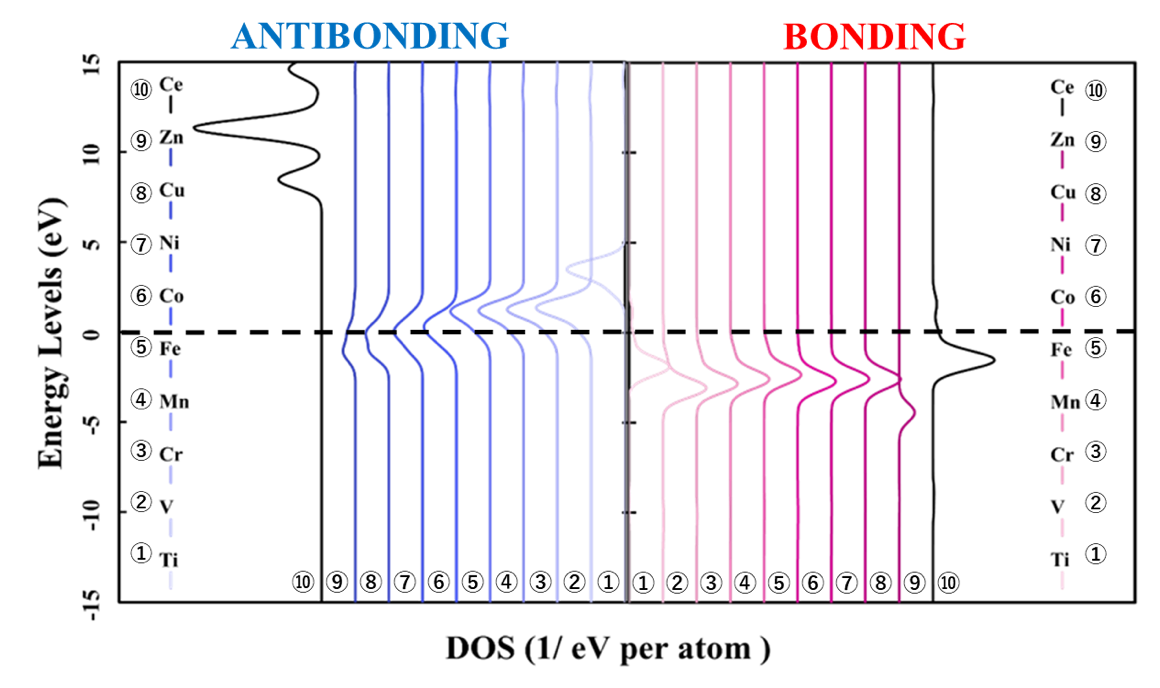


Fig. SI 25. (= Fig. 9) Energy levels of the bond composition between the metal 3d orbitals and the oxygen 2p orbitals with the HOMO level in the M@Ce_12_O_8_^36+^cluster model (M = Ti, V, Cr, Mn, Fe, Co, Ni, Cu, Zn, Ce). In the undoped ceria, the bond composition between the metal 5d orbitals and the oxygen 2p orbitals. The bonding and antibonding orbital components are on the right and left side, respectively.


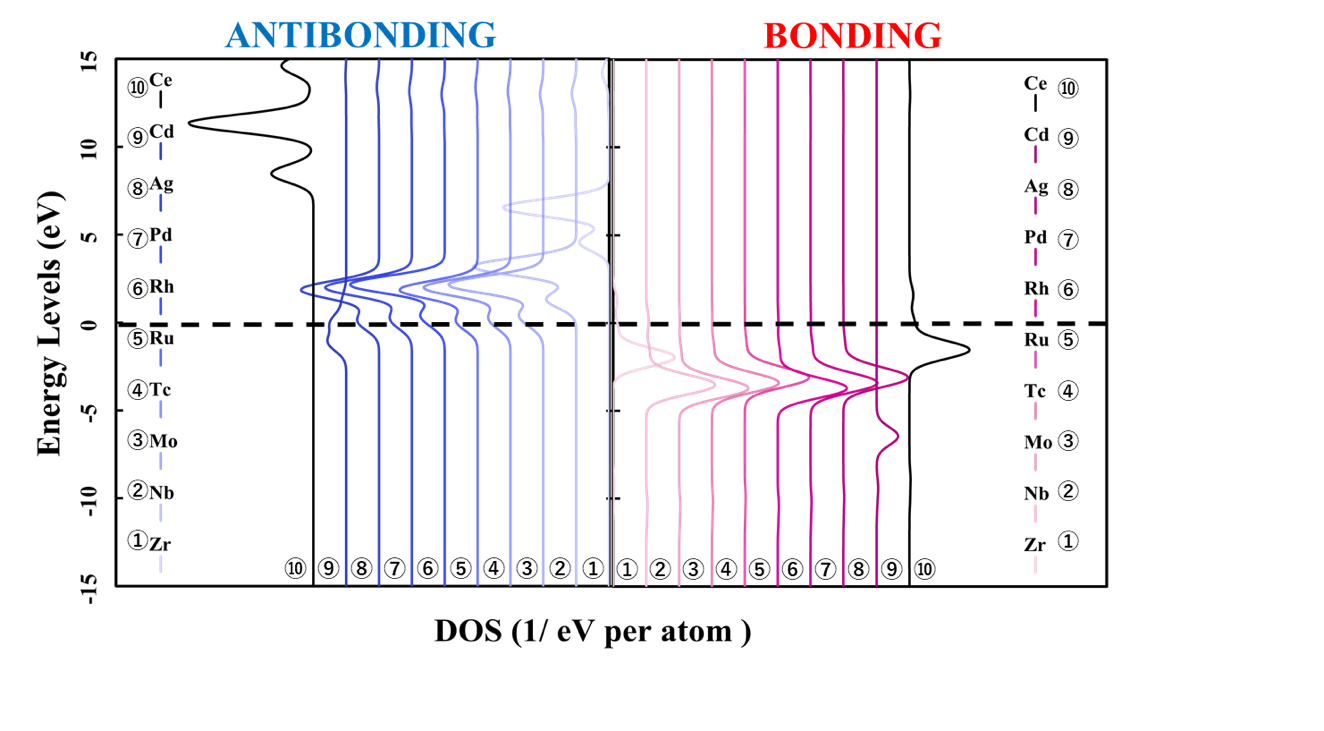


Fig. SI 26. Energy levels of the bond composition between the metal 4d orbitals and the oxygen 2p orbitals with the HOMO level in the M@Ce_12_O_8_^36+^cluster model (M = Zr, Nb, Mo, Tc, Ru, Rh, Pd, Ag, Cd, Ce). In the undoped ceria, the bond composition between the metal 5d orbitals and the oxygen 2p orbitals. The bonding and antibonding orbital components are on the right and left side, respectively.


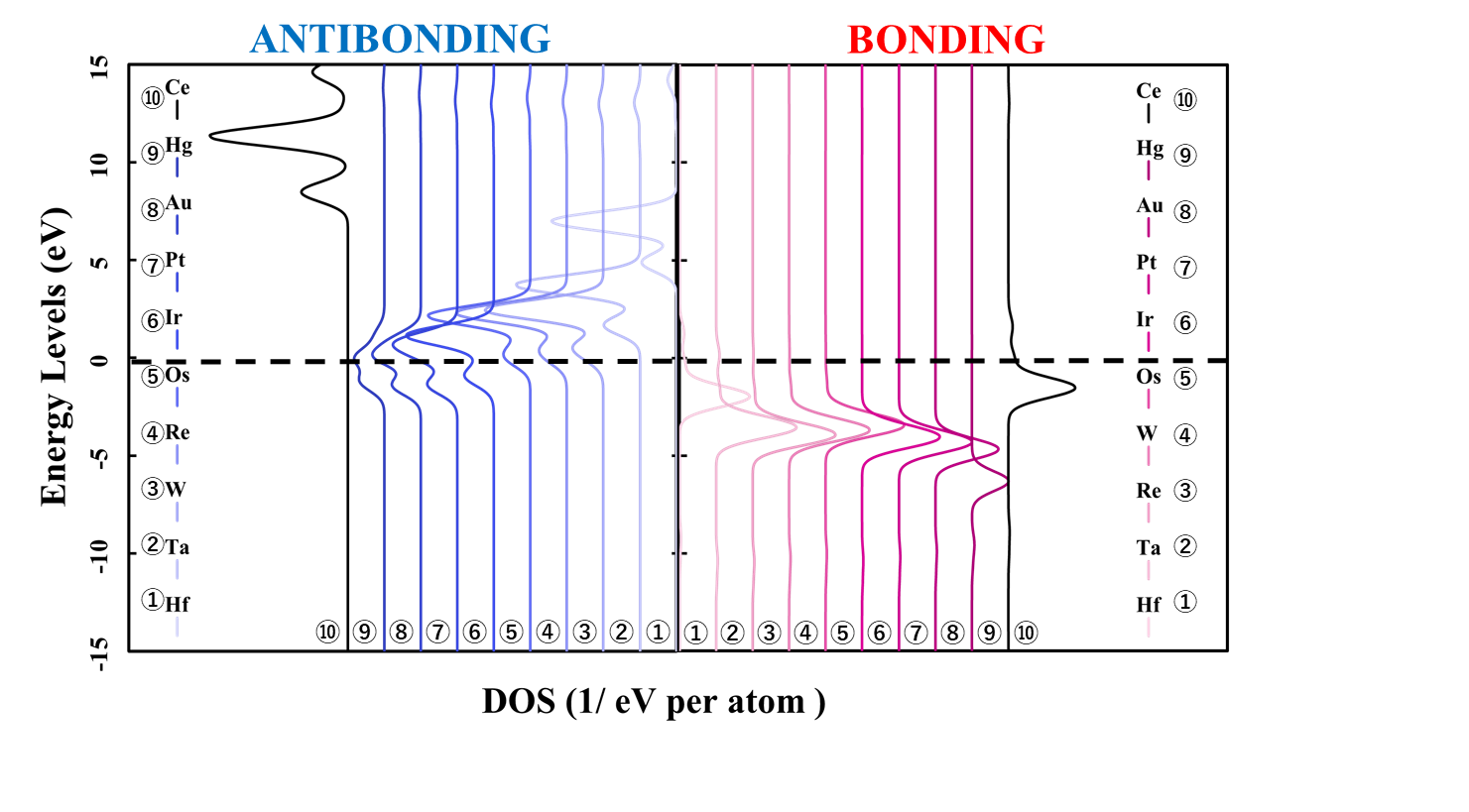


Fig. SI 27. Energy levels of the bond composition between the metal 5d orbitals and the oxygen 2p orbitals with the HOMO level in the M@Ce_12_O_8_^36+^cluster model (M = Hf, Ta, W, Re, Os, Ir, Pt, Au, Hg, Ce). In the undoped ceria, the bond composition between the metal 5d orbitals and the oxygen 2p orbitals. The bonding and antibonding orbital components are on the right and left side, respectively.

**Ti-doped (M = Ti) in the M@Ce_12_O_8_^36+^ cluster model**


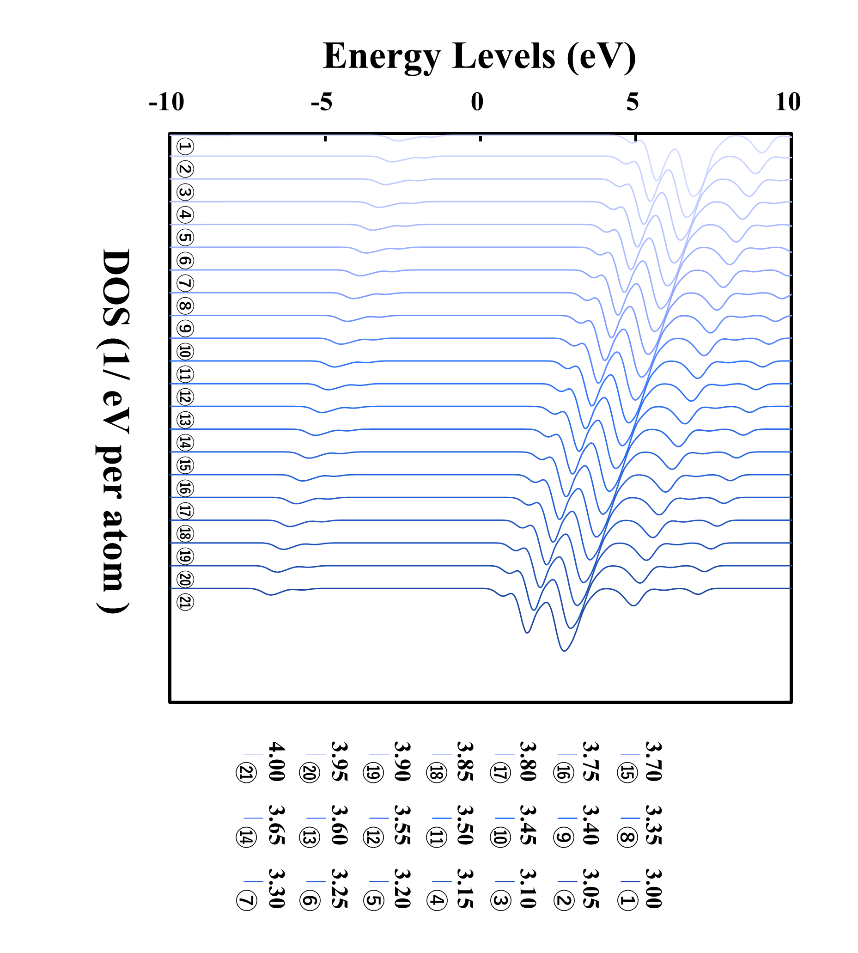


Fig. SI 28. p-DOS for the 2p orbitals of the oxygen atoms in the Ti@Ce_12_O_8_^36+^ cluster model, the vacuum level is the reference energy level.


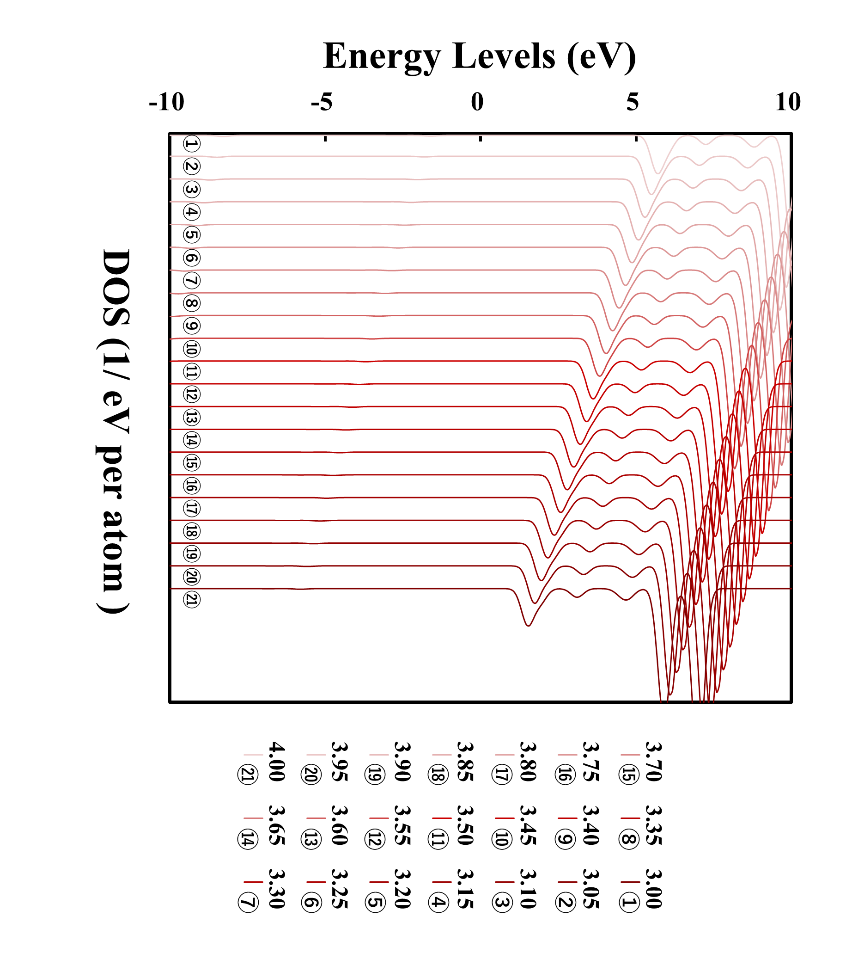


Fig. SI 29. p-DOS for the 3d orbitals of the doped metal atom in the Ti@Ce_12_O_8_^36+^ cluster model, the vacuum level is the reference energy level.

**V-doped (M = V) in the M@Ce_12_O_8_^36+^ cluster model**


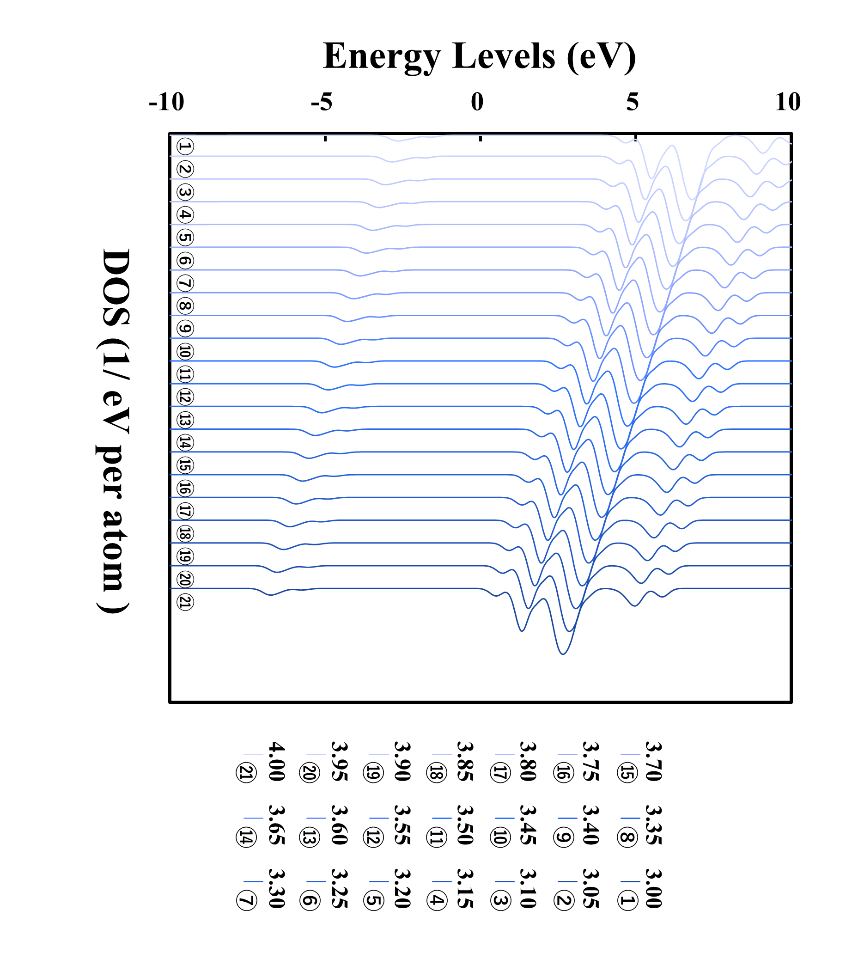


Fig. SI 30. p-DOS for the 2p orbitals of the oxygen atoms in the V@Ce_12_O_8_^36+^ cluster model, the vacuum level is the reference energy level.


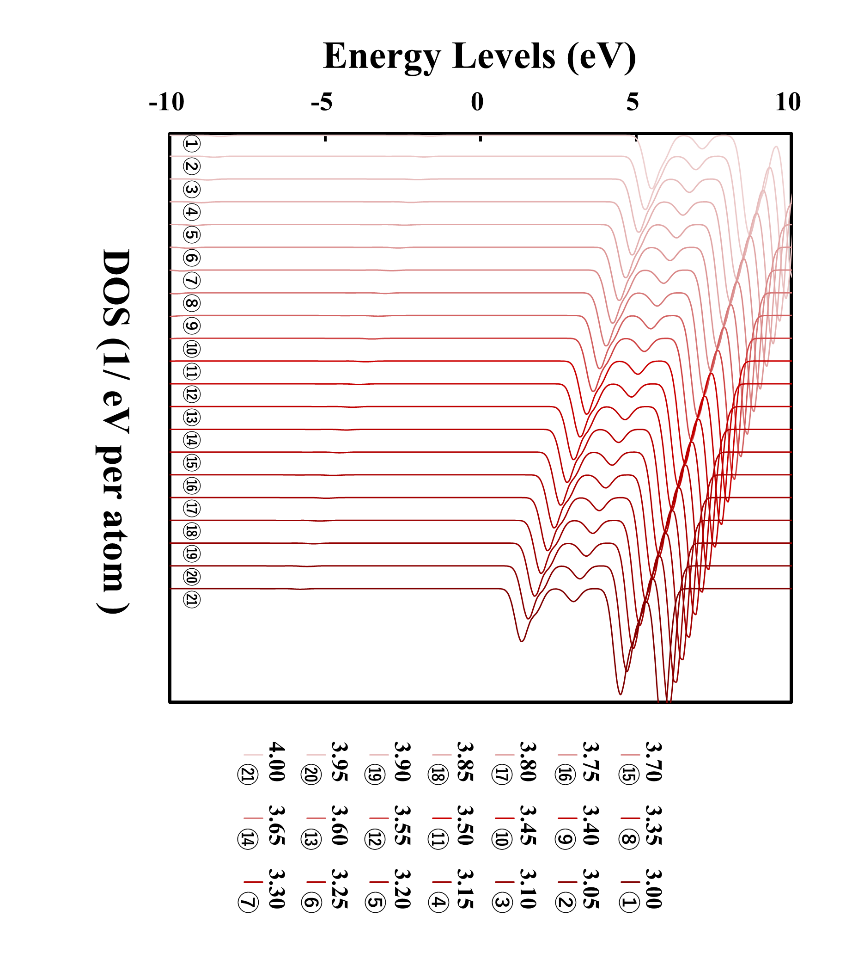


Fig. SI 31. p-DOS for the 3d orbitals of the doped metal atom in the V@Ce_12_O_8_^36+^ cluster model, the vacuum level is the reference energy level.

**Cr-doped (M = V) in the M@Ce_12_O_8_^36+^ cluster model**

**
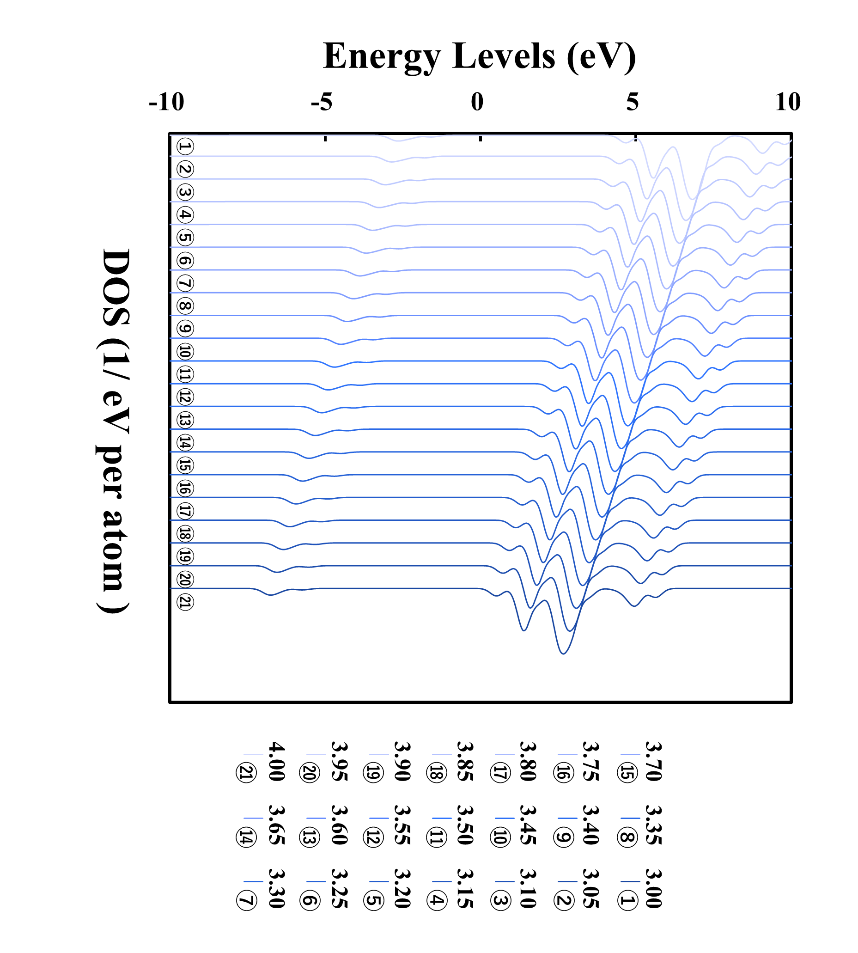
**

Fig. SI 32. p-DOS for the 2p orbitals of the oxygen atoms in the Cr@Ce_12_O_8_^36+^ cluster model, the vacuum level is the reference energy level.

**
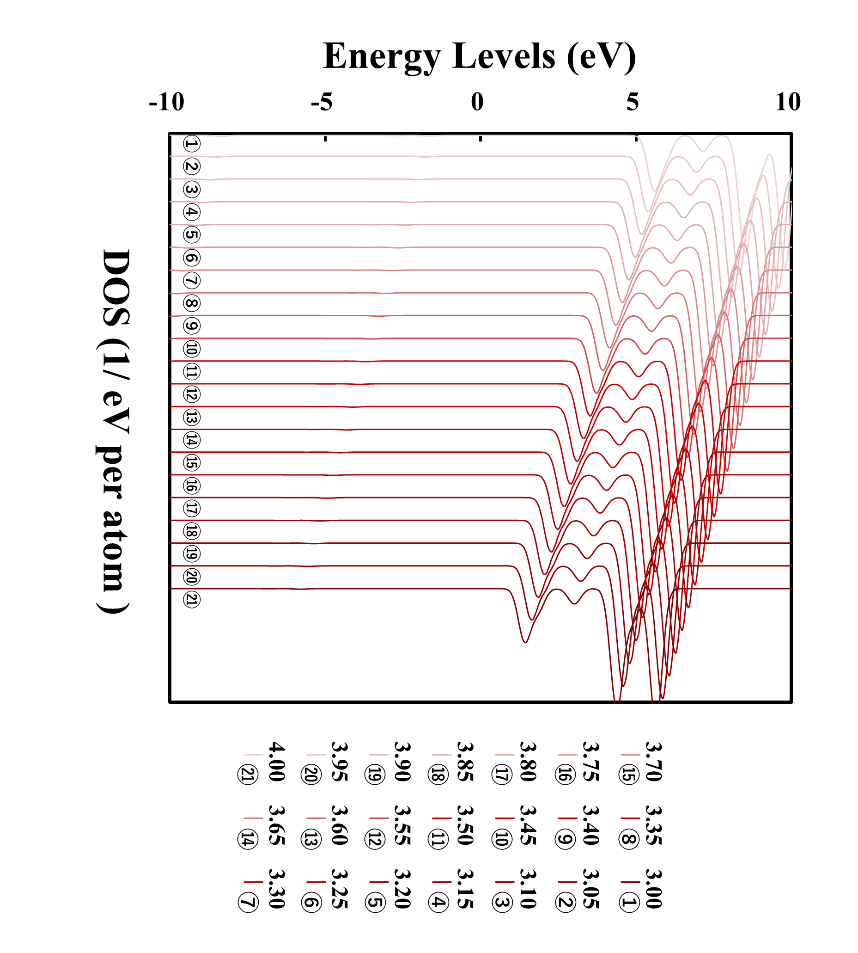
**

Fig. SI 33. p-DOS for the 3d orbitals of the doped metal atom in the Cr@Ce_12_O_8_^36+^ cluster model, the vacuum level is the reference energy level.

**Mn-doped (M = V) in the M@Ce_12_O_8_^36+^ cluster model**

**
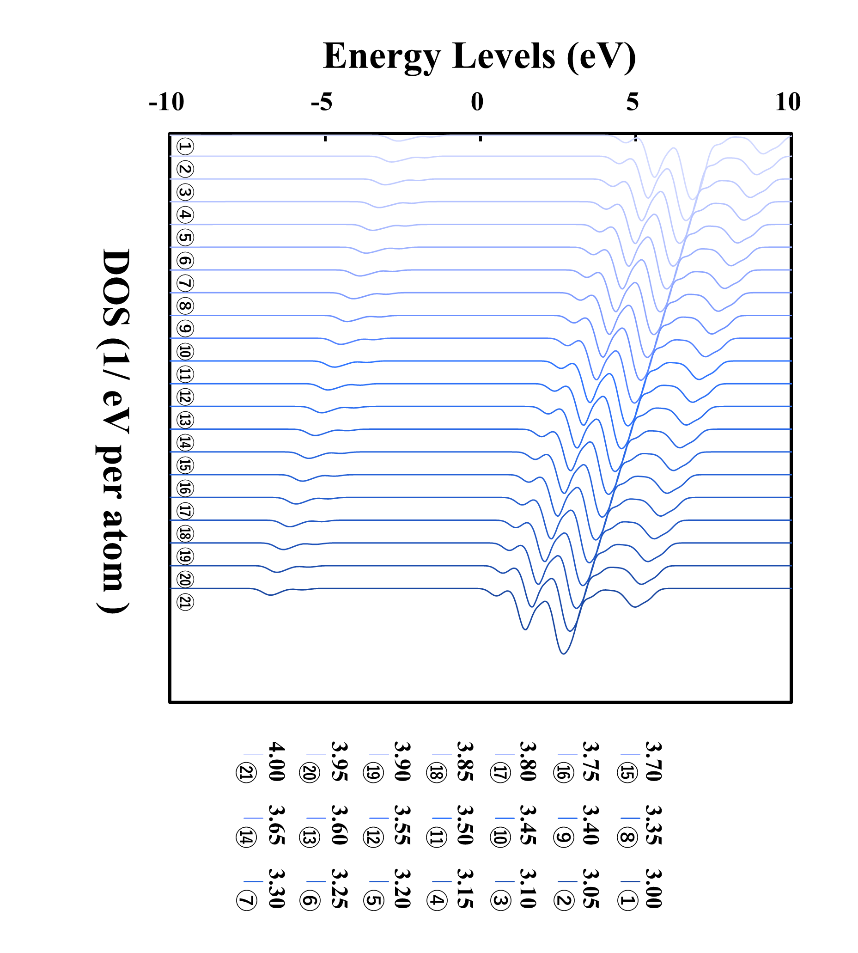
**

Fig. SI 34. p-DOS for the 2p orbitals of the oxygen atoms in the Mn@Ce_12_O_8_^36+^ cluster model, the vacuum level is the reference energy level.

**
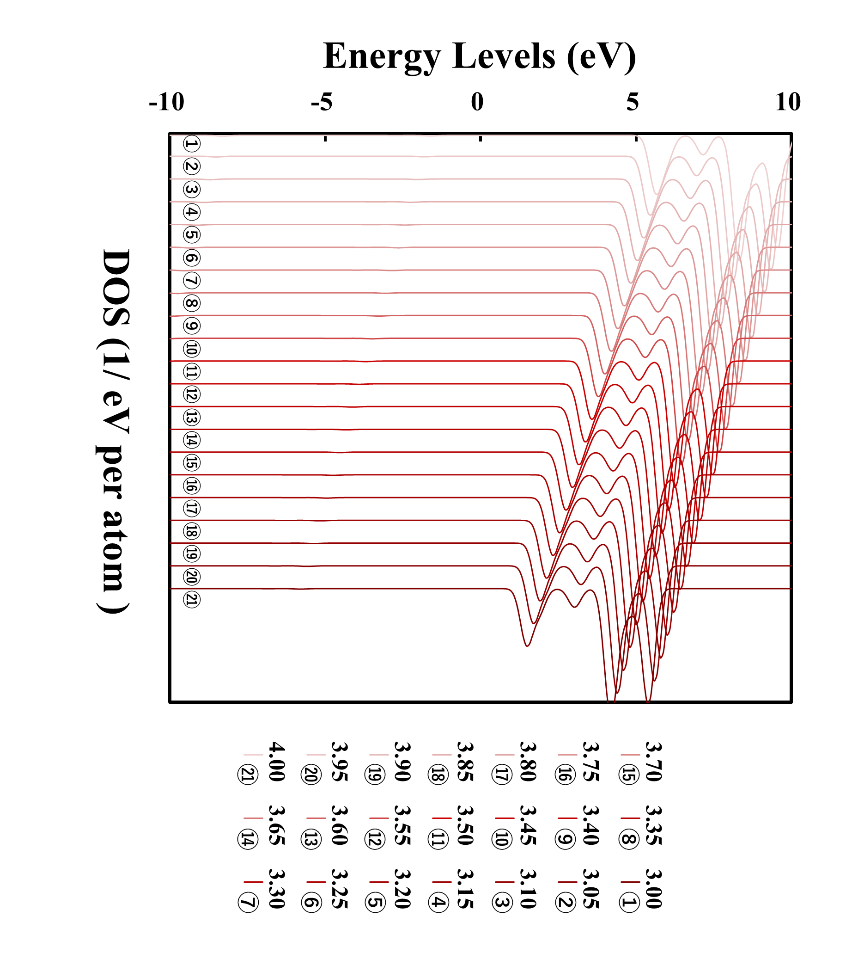
**

Fig. SI 35. p-DOS for the 3d orbitals of the doped metal atom in the Mn@Ce_12_O_8_^36+^ cluster model, the vacuum level is the reference energy level.

**Fe-doped (M = V) in the M@Ce_12_O_8_^36+^ cluster model**

**
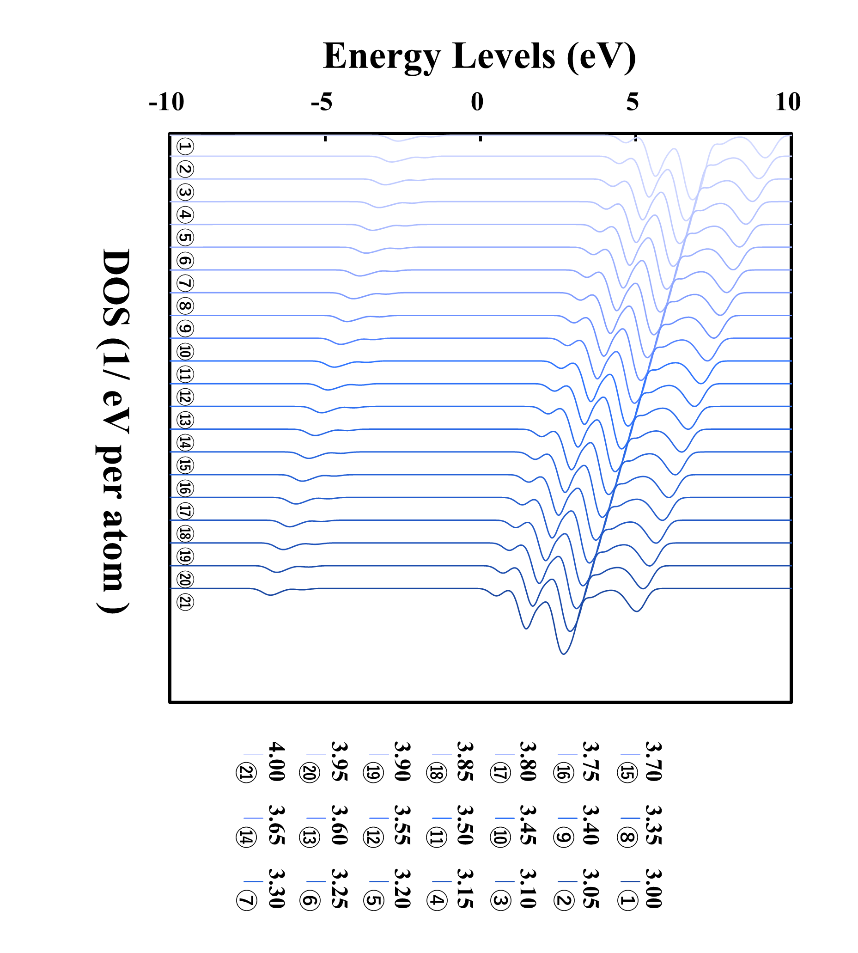
**

Fig. SI 36. p-DOS for the 2p orbitals of the oxygen atoms in the Fe@Ce_12_O_8_^36+^ cluster model, the vacuum level is the reference energy level.

**
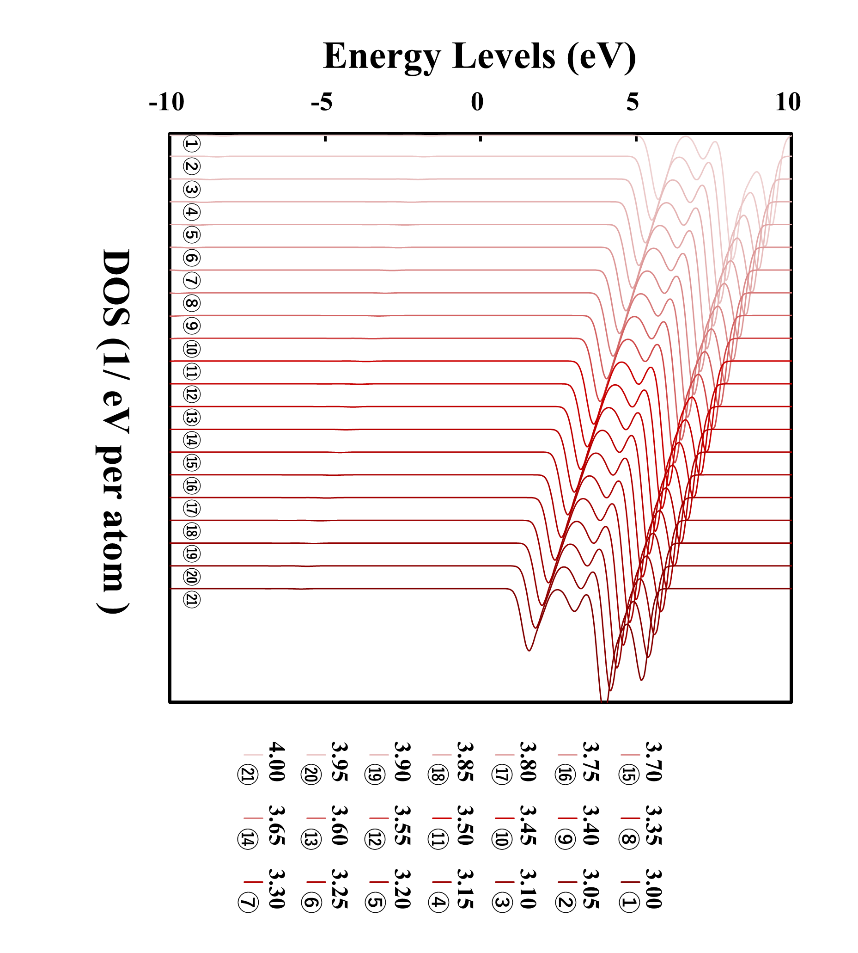
**

Fig. SI 37. p-DOS for the 3d orbitals of the doped metal atom in the Fe@Ce_12_O_8_^36+^ cluster model, the vacuum level is the reference energy level.

**Co-doped (M = V) in the M@Ce_12_O_8_^36+^ cluster model**

**
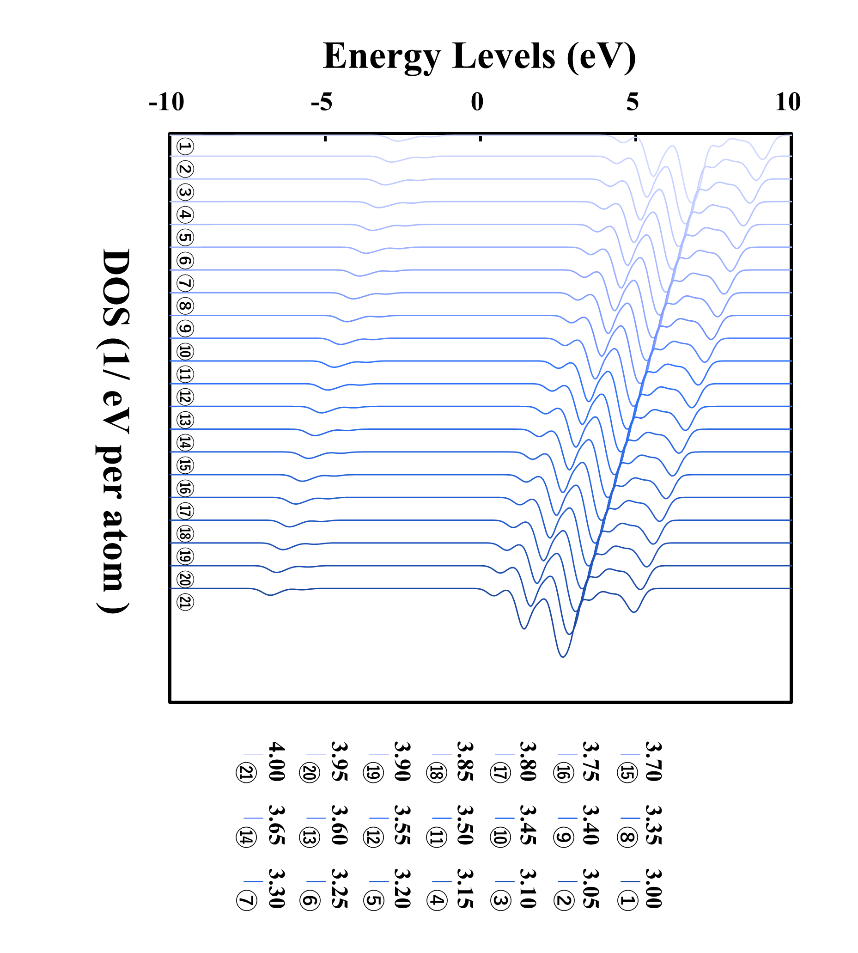
**

Fig. SI 38. p-DOS for the 2p orbitals of the oxygen atoms in the Co@Ce_12_O_8_^36+^ cluster model, the vacuum level is the reference energy level.

**
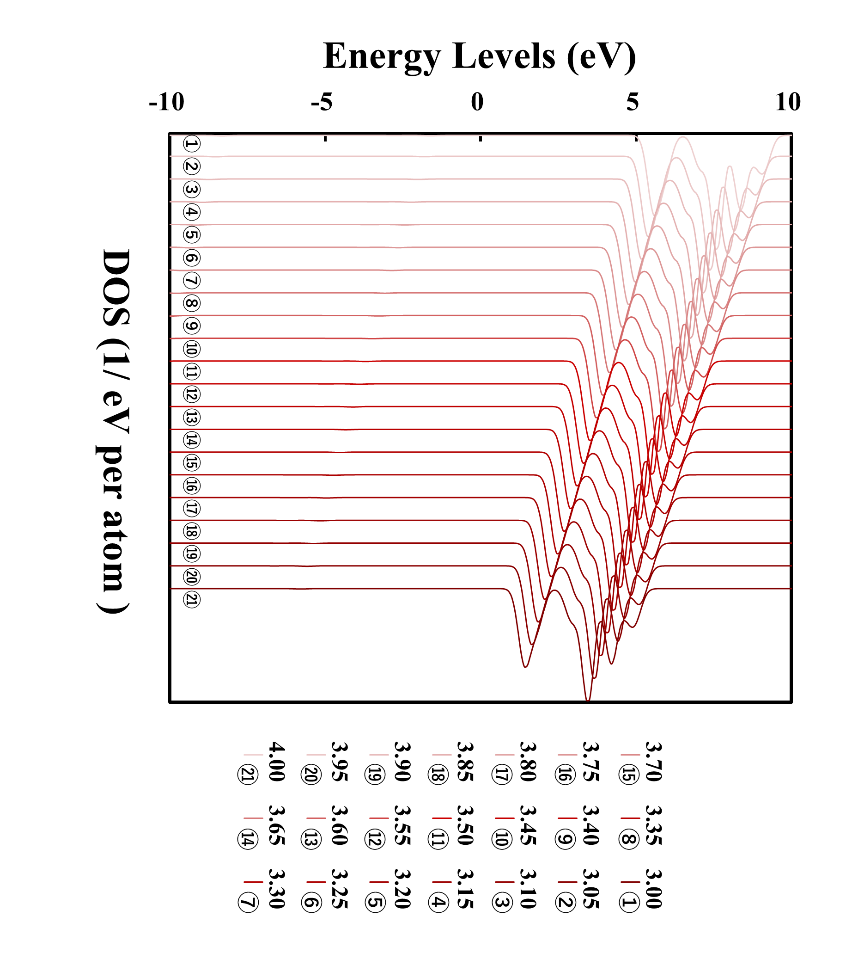
**

Fig. SI 39. p-DOS for the 3d orbitals of the doped metal atom in the Co@Ce_12_O_8_^36+^ cluster model, the vacuum level is the reference energy level.

**Ni-doped (M = V) in the M@Ce_12_O_8_^36+^ cluster model**

**
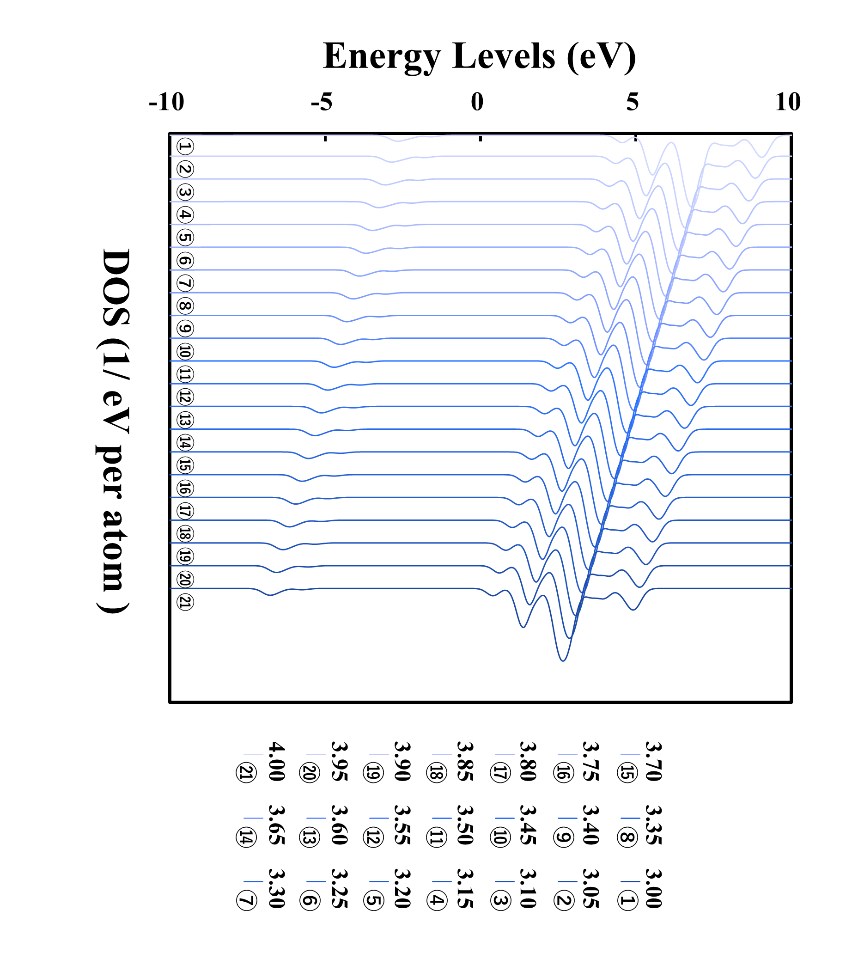
**

Fig. SI 40. p-DOS for the 2p orbitals of the oxygen atoms in the Ni@Ce_12_O_8_^36+^ cluster model, the vacuum level is the reference energy level.

**
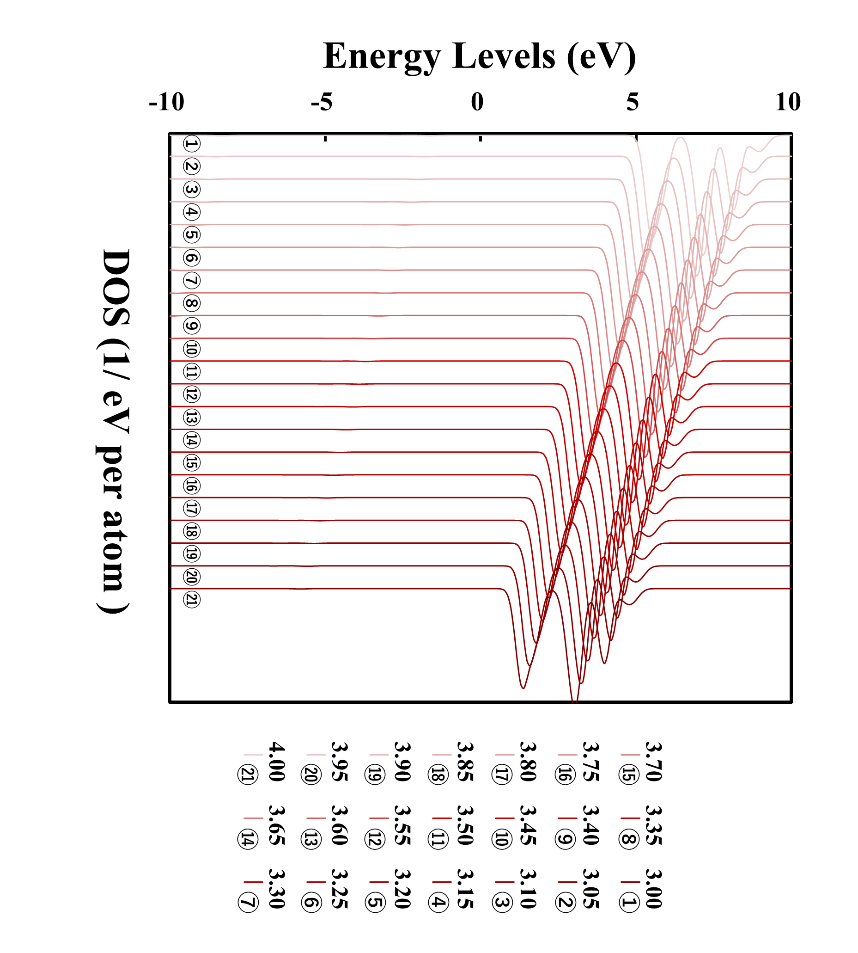
**

Fig. SI 41. p-DOS for the 3d orbitals of the doped metal atom in the Ni@Ce_12_O_8_^36+^ cluster model, the vacuum level is the reference energy level.

**Cu-doped (M = V) in the M@Ce_12_O_8_^36+^ cluster model**

**
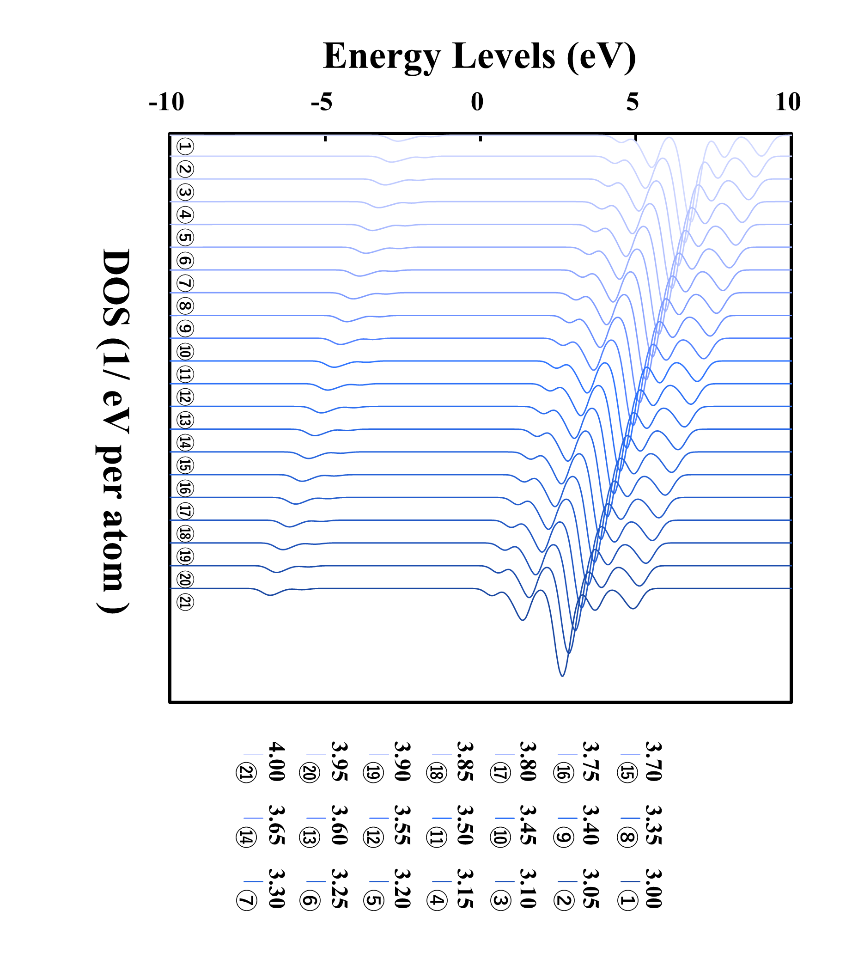
**

Fig. SI 42. p-DOS for the 2p orbitals of the oxygen atoms in the Cu@Ce_12_O_8_^36+^ cluster model, the vacuum level is the reference energy level.

**
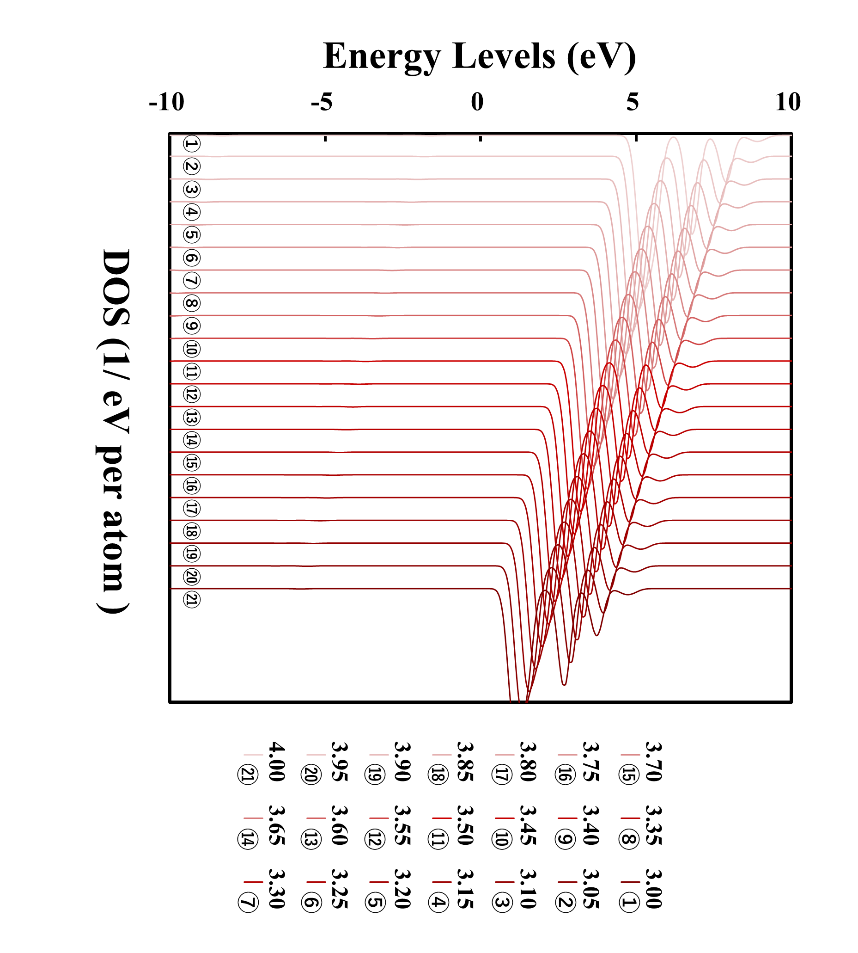
**

Fig. SI 43. p-DOS for the 3d orbitals of the doped metal atom in the Cu@Ce_12_O_8_^36+^ cluster model, the vacuum level is the reference energy level.

**Zn-doped (M = V) in the M@Ce_12_O_8_^36+^ cluster model**


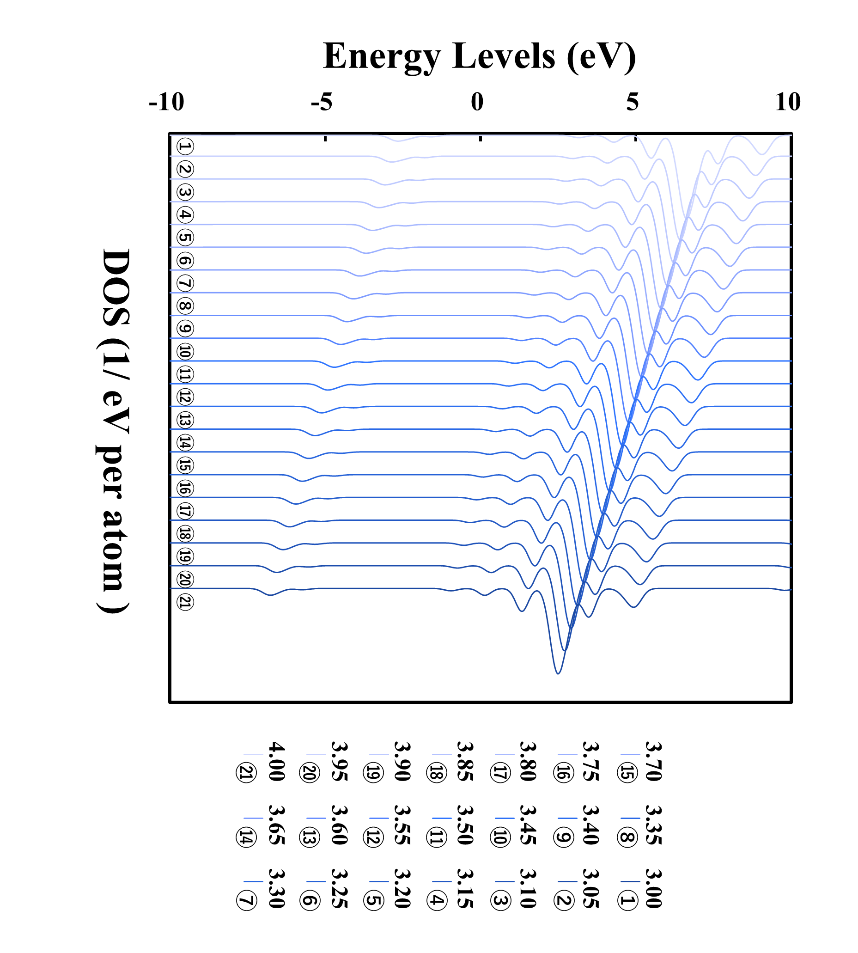


Fig. SI 44. p-DOS for the 2p orbitals of the oxygen atoms in the Zn@Ce_12_O_8_^36+^ cluster model, the vacuum level is the reference energy level.


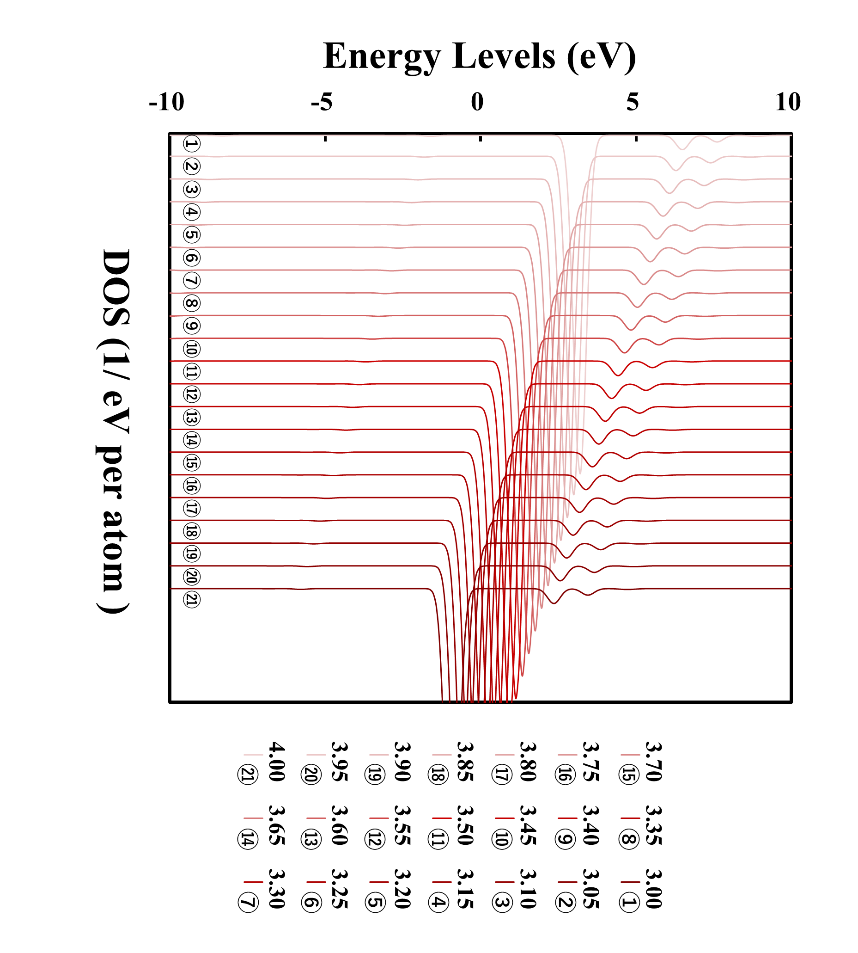


Fig. SI 45. p-DOS for the 3d orbitals of the doped metal atom in the Zn@Ce_12_O_8_^36+^ cluster model, the vacuum level is the reference energy level.

**Pure Ceria (M = Ce) in the M@Ce_12_O_8_^36+^ cluster model**

**
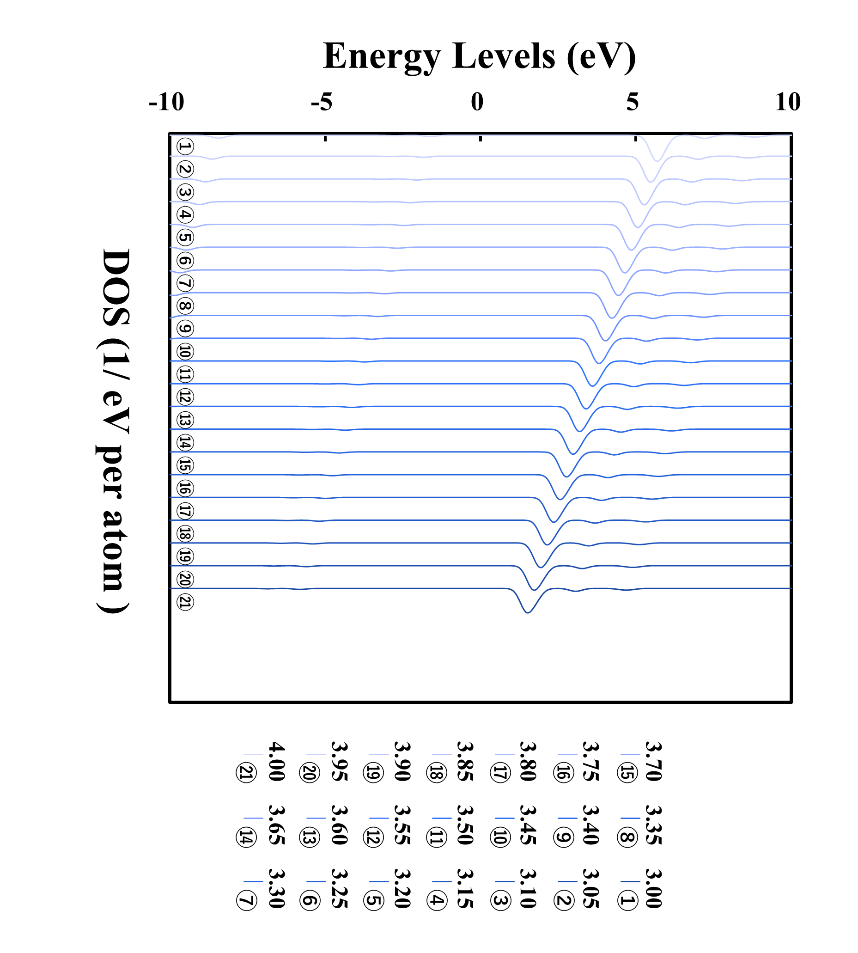
**

Fig. SI 46. p-DOS for the 2p orbitals of the oxygen atoms in the Ce@Ce_12_O_8_^36+^ (Ce_13_O_8_^36+^) cluster model, the vacuum level is the reference energy level.


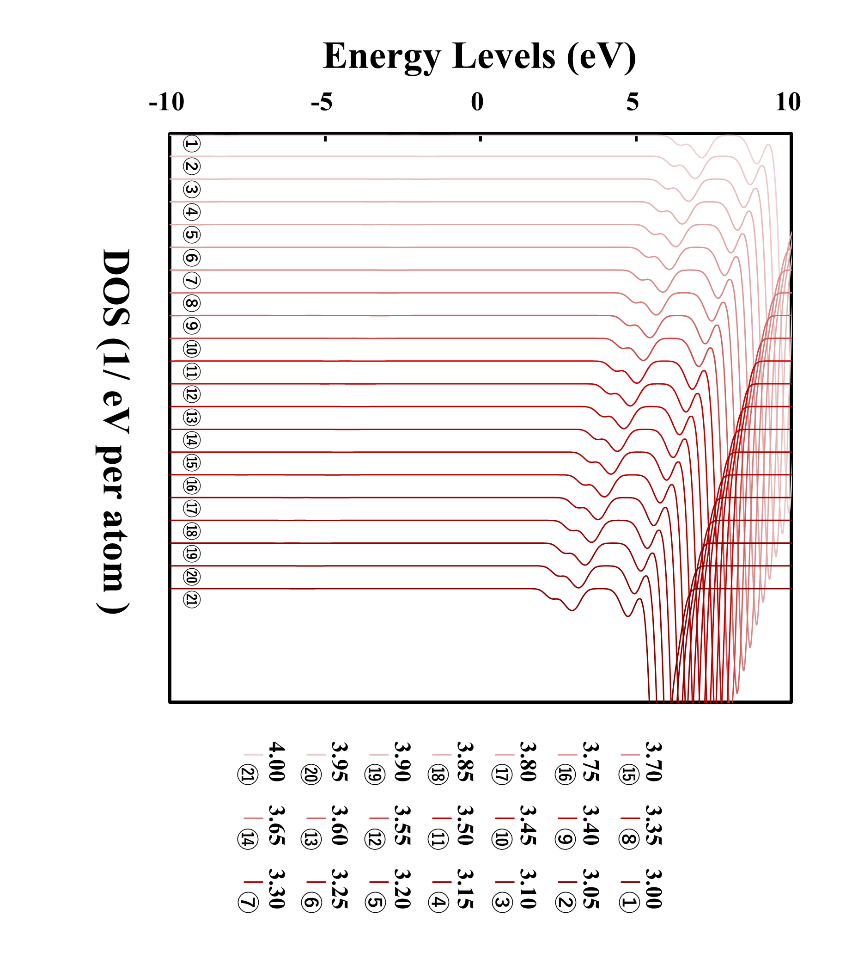


Fig. SI 47. p-DOS for the 5d orbitals of the doped metal atom in the Ce@Ce_12_O_8_^36+^ (Ce_13_O_8_^36+^) cluster model, the vacuum level is the reference energy level.
